# BitterMasS: Predicting Bitterness from Mass Spectra

**DOI:** 10.1021/acs.jafc.3c09767

**Published:** 2024-04-30

**Authors:** Evgenii Ziaikin, Edisson Tello, Devin G. Peterson, Masha Y. Niv

**Affiliations:** † Food Science and Nutrition, The Robert H. Smith Faculty of Agriculture, Food and Environment, The Institute of Biochemistry, Food and Nutrition, The Hebrew University of Jerusalem, 76100 Rehovot, Israel; ‡ Department of Food Science and Technology, College of Food, Agriculture, and Environmental Sciences, The Ohio State University, Columbus, Ohio 43210, United States

**Keywords:** bitterness, classifier, mass spectra, natural products, machine learning, metabolome, taste

## Abstract

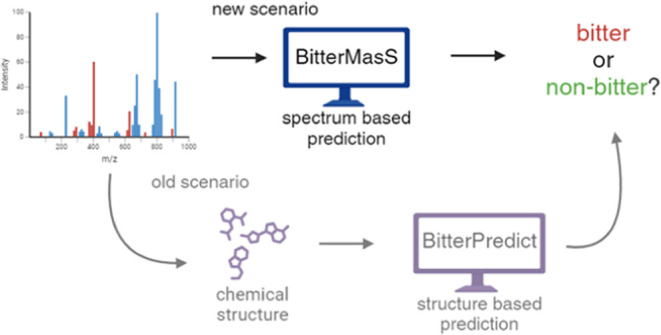

Bitter compounds are common in nature and among drugs. Previously, machine learning tools were developed to predict bitterness from the chemical structure. However, known structures are estimated to represent only 5–10% of the metabolome, and the rest remain unassigned or “dark”. We present BitterMasS, a Random Forest classifier that was trained on 5414 experimental mass spectra of bitter and nonbitter compounds, achieving precision = 0.83 and recall = 0.90 for an internal test set. Next, the model was tested against spectra newly extracted from the literature 106 bitter and nonbitter compounds and for additional spectra measured for 26 compounds. For these external test cases, BitterMasS exhibited 67% precision and 93% recall for the first and 58% accuracy and 99% recall for the second. The spectrum–bitterness prediction strategy was more effective than the spectrum–structure–bitterness prediction strategy and covered more compounds. These encouraging results suggest that BitterMasS can be used to predict bitter compounds in the metabolome without the need for structural assignment of individual molecules. This may enable identification of bitter compounds from metabolomics analyses, for comparing potential bitterness levels obtained by different treatments of samples and for monitoring bitterness changes overtime.

## Introduction

Taste is a pivotal determinant of food preference. Bitter taste is one of the few basic taste modalities sensed by humans.^1^ Together with the sweet and umami modalities, it is mediated by G-protein-coupled receptors (GPCRs), whereas salty and sour modalities are sensed through ion channels.^2−4^ It is broadly assumed that the evolutionary role of bitter taste is to enable animals to avoid consuming toxic food.^5^ However, bitterness is not always a reliable marker of toxicity^6^ and there is evidence for the health benefits of many bitter compounds, such as antioxidant properties and anticancer activity.^7^ Furthermore, while bitterness is a primary deterrent to the consumption of some foods, it is not universally rejected, with typically high acceptance in beverages like coffee, beer, and wine, for which bitterness constitutes a desirable attribute.^7^

In humans, the perception of bitterness is mediated by a family of 25 functional TAS2Rs.^8^ These receptors are expressed in many tissues outside the oral cavity, which may indicate physiological roles beyond food detection and evaluation.^9^ TAS2Rs are already known to mediate hormone secretion^10^ and regulation of innate immunity in the upper respiratory tract.^11^ These discoveries may indicate that new and important biological roles for this family have yet to be discovered.

BitterDB^12,13^ is a database of bitter compounds and associations between bitter molecules and TAS2R subtypes. Currently, it accounts for ∼1040 compounds that cause bitter taste sensation in humans. However, this is only a small fraction of the potential tertiary chemical space. Based on known bitter and nonbitter compounds, machine learning (ML) classifiers were developed, which predict the taste of a molecule based on its chemical structure.^14,15^ BitterPredict estimated that there are at least 11,000 compounds in the ChEBI^16^ database of molecules of biological interest that possess bitterness.^14^ This is still incomparably small compared to the entire chemical space, which is estimated at 10^60^ structures,^17^ with a readily synthetically accessible chemical space of 29 billion compounds and rising rapidly.^18^ The source of the newly discovered bitter molecules may also come from products of natural origin. BitterPredict has previously estimated that more than 70% of the natural compounds possess some bitterness.^14^ Additional ML tools are developed to predict intense bitterness^19^ and the associated TAS2Rs.^20^ These methods use information about the structure of the compound.

Understanding bitterness and identifying bitter compounds are important for several reasons. Bitter taste may be related to toxicity but may also indicate the therapeutic potential of the compound.^6,21^ Exploring predicted bitterness can inform the development of consumer-preferred products and also help find compounds with health benefits, contributing to both product innovation and health.^22^

The identification of small molecules in biological samples is called metabolomics.^23^ One of the main identification tools is mass spectrometry (MS) combined with chromatographic methods, e.g., liquid chromatography (LC) or gas chromatography (GC).^24^ LC/GC-MS is a powerful tool in food analytics; it is used to determine food components^25^ as well as their changes during storage.^26^ In addition, there are applications for LC/GC-MS to optimize the flavor of food products. Usage examples include determination of the change in odor and taste compounds during prolonged natural fermentation,^27^ identification of flavor peptides in beef broth obtained under optimized braising conditions,^28^ etc.^29−31^

Currently, the identification of compounds is mainly performed by comparison with libraries of mass spectra of reference standards. The efficiency of such a strategy, according to various estimates, can range from 5 to 10%,^32,33^ that is, only a small part of the spectra are assigned to known chemical structures, whereas the rest remain unresolved and form the so-called “dark metabolome”.^34^

This is due to the vast structural diversity, high molecular mass, complexity, and unique chemical properties of natural products that together with technical barriers in screening and isolation pose challenges in their identification.^35^ Thus, the identification of new chemical structures of natural products still presents a major challenge.^35^

Tools for assigning mass spectra to chemical structures without involving annotated libraries^33,36,37^ mainly include a combination of deep learning methods and heuristic rules to construct a molecule fitting a given spectrum.^36^ Despite their reasonably good predictive power, this field is still developing. For example, MassGenie^33^ correctly determined the structure of 49 out of 93 compounds (53%) from positive electrospray mass spectra. CFM-ID 4.0,^36^ a tool available in the web server https://cfmid.wishartlab.com, allows both spectral prediction from the structure and compound identification from spectra; the accuracy of chemical structure determination from the spectrum was 71%. CFM-ID 4.0 performance varied depending on collision energy and the compound type, with better MS/MS predictions for benzenoids than for heterocyclic compounds.^38^

In parallel, other methods predict the properties of compounds directly from the mass spectra without the intermediate step of structure determination. Some examples include definition of a class of chemical compounds, CANOPUS tool^39^ (average precision = 99.7%), its toxicity^40^ (*R*^2^ = 0.7 for regression model and balanced accuracy (BA) = 0.7 for classification model), or its odor.^41^ However, to the best of our knowledge, there have been no attempts to predict the taste of a compound from its mass spectrum. If successful, such a tool would allow the detection of taste compounds in food samples without the intermediate identification of their structure.

Here, we develop the BitterMasS tool for predicting the bitterness of a compound using its mass spectral data. Furthermore, we demonstrate that under the common conditions where the chemical structures are unknown, predicting bitterness directly from the spectrum is more efficient than a strategy that requires intermediate annotation of the compound before predicting bitterness from the chemical structure.

## Materials and Methods

### Sets of Bitter and Nonbitter Compounds

BitterDB^12^ containing 1041 compounds was used as the main source of bitter compounds. For nonbitter compounds, the set used earlier for training BitterPredict^14^ was used. Several molecules misclassified as “nonbitter”, such as hydrogen peroxide, hydrogen cyanide, phenylthiourea, and 3-(propan-2-yloxy)-1,2-benzothiazole-1,1-dioxide, were excised. An additional source of compounds with labels “bitter” or “not bitter” was taken from the BitterIntense training set,^19^ adding 145 bitter and 75 nonbitter compounds.

Novel TAS2R14 agonists from recent publications were also included.^42,43^ Due to the fact that the activation of only one TAS2R14 receptor was investigated, those compounds that have not been experimentally confirmed as agonists cannot be labeled as “nonbitter” since they may activate other TAS2Rs. Thus, 261 bitter compounds that are TAS2R14 agonists were added.

An additional 20 basic amino acids were also included.^44^ This resulted in 15 amino acids with a “bitter” label and 5 with a “nonbitter” label.

Duplicates were identified and removed based on InChIKey, which was generated using RDKit.^45^ The full length of InChIKey, which includes information about the binding, stereochemistry, and protonation of the molecule, was used. The final set of bitter and nonbitter compounds included 1418 bitter and 1579 nonbitter compounds. When combined, the resulting dataset contained 2511 labeled molecules.

### Mass Spectral Data

Mass spectral data were searched in MassBank^46^ (September 1, 2023). MassBank is a publicly available repository of mass spectral data for aqueous small chemical compounds. It serves as a resource for researchers in the field of mass spectrometry, allowing them to compare and identify unknown compounds by comparing their mass spectra with the reference spectra in the database.

The database was chosen because it is open-source, making it easy to deploy locally. We also downloaded data from the MassBank of North America (MoNA),^47^ which includes several mass spectral databases, including GNPS^48^ (Table S1). Additional data were obtained for 26 bitter and 26 nonbitter compounds, corresponding to 262 and 341 additional spectra, respectively. The models trained with these additional data had the same performance as models trained on MassBank alone. Therefore, MassBank was retained as a source of the experimental mass spectra.

The search was performed locally in a cloned repository by using InChIKey. The SMILES, MS type, ionization type, ionization energy, and *m*/*z* data and their intensities were extracted. Intensity values were converted by min–max normalization to values between 0 and 100. Only electron ionization mass spectrometry (EI-MS) and electron ionization tandem mass spectrometry (ESI-MS/MS) spectra in positive ion mode were extracted. This resulted in three sets of spectra: the EI-MS set included 1533 spectra for 667 compounds; the ESI-MS/MS set included 3881 spectra for 312 compounds; and a combined set was obtained by merging the previous two (Table 1).

**Table 1:** Number of Mass Spectra for Bitter and Nonbitter Compounds in the Prepared Datasets

	number of compounds		number of spectra	
dataset	bitter	nonbitter	bitter	nonbitter
EI-MS	173	494	413	1120
ESI-MS/MS	244	68	3164	717
combined dataset	348	512	3577	1837

### Splitting into Training and Test Sets

Multiple mass spectra can be obtained for a single compound. In addition, spectra could have been obtained using different instrumentations, collision energies, and fragmentation processes. Due to the many variables, both very similar and very different spectra can be found for a single compound (Figure S2). When splitting the training and test sets by spectra, similar spectra may appear in both sets, which will inevitably lead to overestimation of the model during validation. For this reason, the split of the set into training and test was done by compounds rather than by spectra.

These spectra may or may not be similar to each other. An example compound is antipurine, also known as phenazone, which is an analgesic, fever-reducing, and anti-inflammatory drug (InChIKey = VEQOALNAAJBPNY-UHFFFAOYSA-N), for which the largest number of spectra from different sources was found (Figure S1A).

We selected 80% of bitter and nonbitter compounds to be used as a training set and the remaining 20% to be used as a test set. This sampling process was repeated 20 times randomly. Next, the molecules in the training and test sets were replaced with all of the corresponding mass spectra for each compound. Thus, all of the available spectra for a compound were placed in either a training or test set.

### Calculation of Descriptors

The generated descriptors consisted of two parts. The first one included information about the instrument with which the spectrum was obtained, such as the MS type, ionization type, and energy of ionization. The MS type and ionization type were coded by using natural numbers. Energy of ionization was coded using the corresponding energy value measured in eV. If the ionization energy was not known for the spectrum, then, the corresponding value was set to zero.

The second part of the descriptors included information about peaks present in the spectrum, for generation of which, a binning strategy was used. Each bin represented an interval of *m*/*z* values. The bin value contained information about the peaks that fell within the interval. Bins were generated based on the minimum and maximum *m*/*z* values in the training set, and their increase was equal to the initially set value.

Three strategies for generating mass spectral descriptors were investigated. The first strategy, the bin values, were the total intensities of the peaks falling within the interval. In case the obtained descriptor vector had values exceeding 100, the vector was additionally subjected to min–max normalization, and its values were reduced to the interval from 0 to 100.

In the second strategy, the values of bins were ≪zero≫ if no peak falls into this interval and ≪one≫ if at least one peak falls into this interval.

The third strategy used the number of peaks falling into this interval as the bin value.

Those bins that did not contain a single peak during training were removed and were not considered by the model in the future. Next, instrumental and mass spectral descriptors were concatenated into one vector.

### Training and Optimization of Model Hyperparameters

In this paper, we tested two machine learning algorithms, Random Forest^49^ and XGBoost.^50^ Both methods are based on ensembles of decision trees, but XGBoost has a more modern implementation by using gradient boosting.

Models were trained using the training set and validated on the test set. To fine-tune models for predicting bitterness, we implemented hyperparameter optimization via grid search, supplemented by fivefold cross-validation. The specific hyperparameter values engaged in this optimization can be found in Tables S2 and S3. Given the imbalanced nature of our mass spectral dataset, balanced accuracy served as the chosen optimization metric. For example, in the EI-MS set, nonbitter mass spectra prevailed (707 spectra), while in the ESI-MS/MS set, on the opposite, bitter mass spectra prevailed (2447 spectra). In the combined set, however, the imbalance is slightly reduced, but the compounds are dominated by nonbitter ones (164 compounds), while the mass spectra are dominated by bitter ones (1740 spectra).

### Model Evaluation Metrics

The metrics used to evaluate the effectiveness of the model were precision, recall, balanced accuracy, and the F1-score. These metrics were calculated based on four outcomes in data classification: true positives (TP), true negatives (TN), false positives (FP), and false negatives (FN). Accuracy represents the proportion of correctly predicted positive instances (eq 1). Recall estimates the model’s ability to identify all positive instances by calculating the proportion of correctly predicted positive instances from all actual positive instances (eq 2). The F1-score is a weighted average of the precision and recall (eq 3).

The balanced accuracy was calculated as a metric for the model in this study (eq 4). Selectivity determines how well the model can identify true results (eq 5). Accuracy is a measure of how accurately the model predicts the correct answers (eq 6).
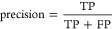
1
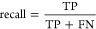
2
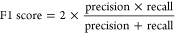
3
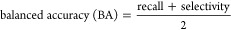
4
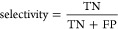
5
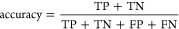
6

For comparison, a naive model that predicts spectra based on the proportion of bitter and nonbitter spectra in the training set was used. Namely, the naïve model predicts that any spectrum belongs to a bitter compound with 0.66 probability and nonbitter with 0.34 probability.

### Literature Search for Obtaining External Test Set 1

To verify the performance of BitterMasS, an external set was extracted from the literature following model training. This external set was prepared by analyzing sensory information on compounds from 18 papers.^51−52,53,60,61,68^ Structures of the compounds were extracted from the articles as follows. If the name of the molecule was available, a search in the PubChem^69^ chemical compound database was performed followed by SMILES extraction. If the search was inconclusive, MarvinSketch^70^ was used to manually draw and extract SMILES. The compounds were extracted along with their respective sensory labels. If the sensory label contained information about the bitterness of a compound, it was labeled as “bitter.” In other cases, compounds were labeled “nonbitter”. As a result, 870 compounds (447 bitter and 375 nonbitter) were extracted in the SMILES format.

Unfortunately, mass spectra were not deposited with the papers directly. For this reason, the experimental spectra for the external set compounds were searched in MassBank^46^ in the same way as in the case of the training set. DrugBank^71^ was also used as an additional source of experimental mass spectra for the medication^52^ and vitamin^56^ categories. As a result, 759 spectra were retrieved for 106 compounds (393 spectra for 67 bitter compounds and 366 spectra for 39 nonbitter compounds). The number of compounds with sensory labels extracted for the external set and the number of spectra found for these compounds are listed in Table 2.

**Table 2:** Number of Bitter and Nonbitter Compounds Extracted for the External Set with the Number of EI-MS and ESI-MS/MS Mass Spectra Found in MassBank and DrugBank^a^

	no. of compounds (no. of compounds with available spectra)		no. of spectra	
source (year) (reference no.)	bitter	nonbitter	bitter	nonbitter
peptides (2023)^51^	320 (6)	320 (2)	19	24
medications (2002)^52^	37 (35)	14 (13)	177	108
kimchi (2022)^53^	4 (0)	16 (4)		24
oat (2020)^54^	17 (2)		7	
wheat bran (2022)^55^	16 (4)		15	
vitamins (2022)^56^	4 (3)	9 (7)	3	95
olive oil (2006)^57^	9 (1)		2	
bitter odorants (2023)^58^	7 (2)	1 (0)	2	
bile acids (2023)^59^	8 (7)		32	
wheat bread (2013)^60^	6 (1)		1	
sugars (2019)^61^		6 (5)		21
dry-hopped beer (2022)^62^	6 (2)		35	
soy sauce (2022)^63^	4 (1)	1 (1)	18	22
hazelnuts (2021)^64^	4 (1)		19	
polyphenols (2013)^65^	4 (1)		35	
green tea (2020)^66^		3 (2)		12
citrus fruit (2013)^67^	1 (1)	2 (2)	28	36
bread crumb (2022)^68^		3 (3)		24

a The number of compounds for which at least one EI-MS or ESI-MS/MS spectrum could be found is given in brackets.

### Laboratory Measurements for External Test Set 2

#### Chemicals

LC-MS-grade solvents and additives (methanol, acetonitrile, and formic acid) were purchased from Fisher Scientific (Fair Lawn). Analytical standard alkyl parabens were obtained from Sigma-Aldrich (St. Louis). Nanopure water was obtained by using a water purification system (Barnstead Nanopure Diamond, Thermo Scientific, Dubuque, IA). Cis-isocohumulone, Cis-iso-*N*-humulone, Cis-iso-adhumulone, α-chaconine, α-solanine, inosine monophosphate, adenosine monophosphate, uridine monophosphate, and guanosine monophosphate were purchased from BOC Sciences (New York). 3-O-Caffeoylquinic acid, 4-caffeoylquinic acid, 5-caffeoylquinic acid, caffeic acid, genistein, daidzein, and soyasaponin were purchased from Sigma-Aldrich (St. Louis). Monosodium glutamate was purchased from Fisher (Fair Lawn). Pyroglutamic acid was purchased from Akos (Lörrach, Germany). Rebaudioside A at 98% purity was purchased from the Tokyo Chemical Industry (Portland, OR). 2-(3-Hydroxy-2-oxoindolin-3-l) acetic acid 3-O-6′-glucopyranosyl-2″-(2″oxoindolin-3″yl) acetate (Isomer 1), 2-(3-hydroxy-2-oxoindolin-3-l) acetic acid 3-O-6′- glucopyranosyl-2″-(2″oxoindolin-3″yl) acetate (Isomer 2), 1″-O-3′-b-glucofuranosyl-1′-O-1-b-glucofuranosyl-(2,6-dihydroxyphenyl)-ethan-4-one, 1′′-O-1′-b-glucofuranosyl-9-O-6′-b-glucopyranosyl-2″-(2″oxoindolin-3″yl) acetate (Isomer 1) and 1″-O-1′-b-glucofuranosyl-9-O-6′-b-glucopyranosyl-2″-(2″oxoindolin-3″yl) acetate (Isomer 2), rebaudioside A degradation compound 1, rebaudioside A degradation compound 2, rebaudioside A degradation compound 3, and 3-O-caffeoyl-4-O-3-methylbutanoyl quinic acid were obtained from Flavor Research and Education Center, Ohio State University, OH.

#### Sample Preparation

The 15 selected compounds were weighed separately and diluted in 1:1 (v/v) methanol/water according to their solubility. Individual stock solutions were prepared at a concentration of 1 mg/mL and diluted to 5 mg/L working standards for LC-MS analysis.

#### Liquid Chromatography/High-Resolution Mass Spectrometry (HR-MS)

Mass fragmentation analysis of selected compounds was conducted with an Agilent 1290 Infinity II LC system with a binary high-speed pump, sample manager, multicolumn thermostat, and diode array detector coupled with an Agilent 6546 QTOF mass spectrometer (Agilent Technologies, Santa Clara, CA). 1 μL of samples was injected on an Acquity UPLC HSST3 (2.1 × 100 mm^2^, 1.8 μm, Waters Co., Milford, MA) column operated at a flow rate of 0.5 mL/min. The binary-solvent system consisted of water (A) and acetonitrile (B), both containing 0.1% formic acid at a gradient as follows: 0–0.5 min, 0% B; 0.5–9 min, 100% B; and 9–11 min, holding 100% B. The column was re-equilibrated for 2 min at initial conditions between injections, and column temperature was maintained at 40 °C for the entire analysis. The typical operating source conditions for MS and MS/MS scans were optimized in positive mode of ESI as follows: mass scan range = 100–1700 Da, two spectra per second for both MS and MSMS parameters, capillary voltage = 3500 V, nozzle voltage = 1000 V, desolvation temperature = 320 °C, desolvation gas flow rate = 10 L/min, sheath gas temperature = 350 °C and sheath gas flow rate = 11 L/min, fragmentor voltage = 175 V, and skimmer voltage = 65 V. Several collision energies ranging 5 to 70 eV were recorded with an acquisition time of 500 MS/spectrum for both MS and MS/MS parameters. Reference masses to check mass accuracy through the entire analysis were *m*/*z* 121.0508 and *m*/*z* 922.0098 for positive ionization mode. Data acquisition was performed using Mass Hunter workstation software (Version 10.0).

#### External Test Set 2 from Laboratory Measurements

Laboratory measurements were used as external set 2. The compound identification was performed by ESI-MS/MS mass spectrometry, followed by bitterness sensory evaluation. In this way, 111 experimental mass spectra for 26 compounds were obtained (73 spectra for 15 bitter compounds and 38 spectra for 11 nonbitter compounds).

## Results

### Distribution of Mass Spectra in the Datasets

**Figure 1:**
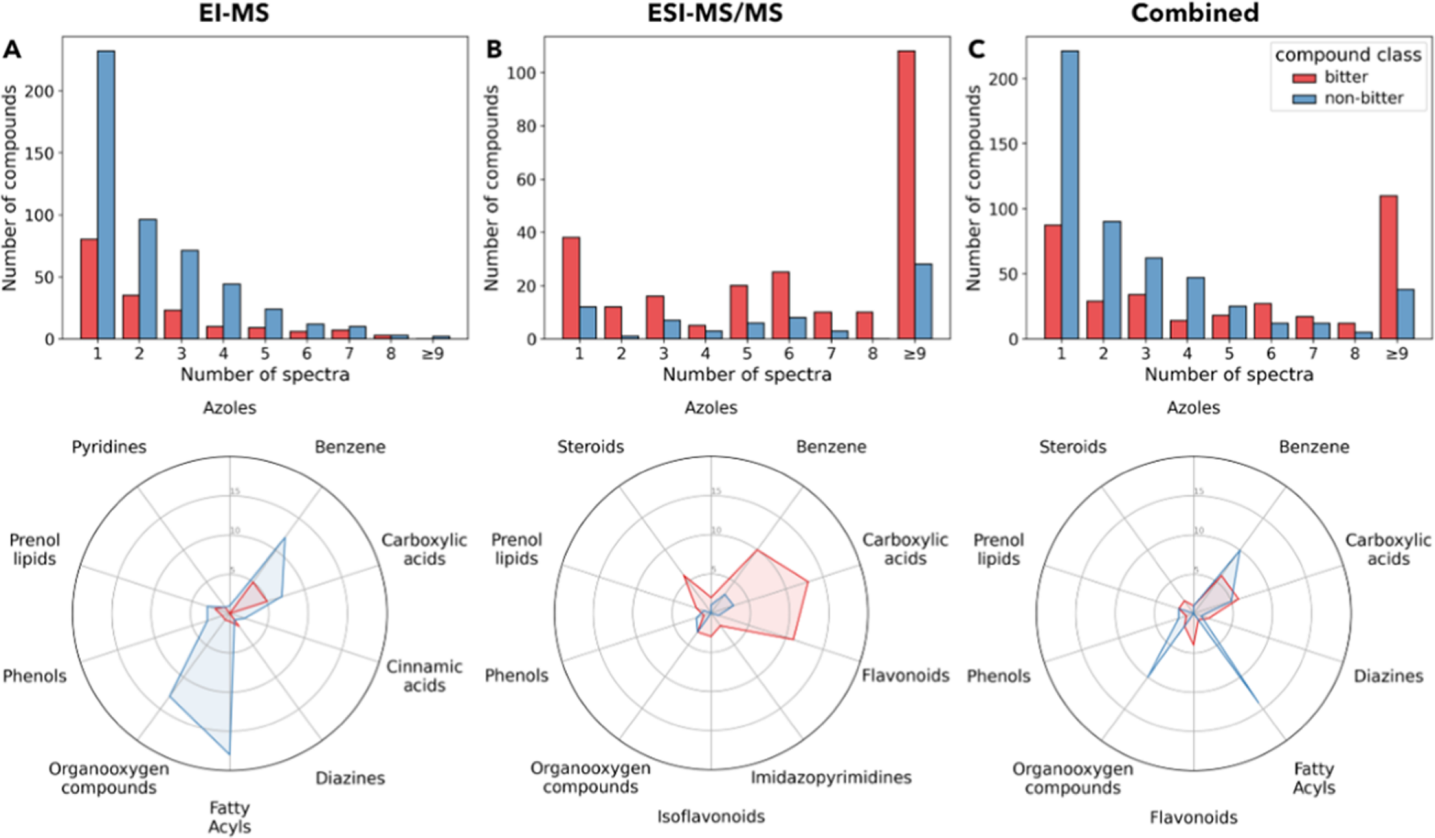
Distributions of the number of mass spectra per compound and the top-10 most popular chemotypes in EI-MS (A), ESI-MS/MS (B), and combined (C) datasets.

Three sets of mass spectra were prepared to train the BitterMasS model: EI-MS, ESI-MS/MS, and a combined set based on the previous two. EI-MS and ESI-MS/MS sets are initially not balanced in the number of bitter vs nonbitter spectra. Moreover, in the case of the EI-MS set, nonbitter spectra predominate, while in the case of the ESI-MS/MS set, bitter spectra predominate. Furthermore, in the case of the EI-MS set, predominantly, one spectrum per compound is found (Figure 1A). The number of compounds for which more than one spectrum are found decreases with increasing number of spectra found. In the case of the ESI-MS/MS set, for most of the compounds, nine or more spectra are found, and these are mostly spectra related to bitter compounds (Figure 1B). Combining the EI-MS and ESI-MS/MS sets somewhat eliminates this imbalance, but there is still a surplus of bitter spectra (Figure 1C).

This situation creates a certain difficulty in dividing the set into training and test sets based on mass spectra only. In a random separation, the spectra for the same compound can end up in both sets. This in turn can lead to overestimated results when testing the model on the test set, given the assumption that the spectra for the same compound are similar. This is not always the case (see examples and discussion in the Supporting Information); the splitting into training and test was based on compounds (with all their spectra) rather than on individual spectra.

### Development of BitterMasS

During model development, several choices had to be made regarding the training and test datasets, the machine learning algorithm, the bin size, and the way peak descriptors were represented. The estimates used to select these parameters are described below.

#### Selection of the Dataset and Machine Learning Algorithm

To estimate which mass spectral dataset and which machine learning algorithm to use for bitterness prediction, six models with optimized hyperparameters (three Random Forest models and three XGBoost models) were trained and tested. The bin size was set to 0.1 *m*/*z*, and the type of peak descriptors was set based on intensity (see the Supporting Information).

The combined dataset that used both EI-MS and ESI-MS/MS data performed by far better than the separate sets, on both the training and internal test sets. For both Random Forest and XGBoost, the metric values are almost identical, with a slight advantage in favor of Random Forest at 0.01. This may indicate that combining the EI-MS and ESI-MS/MS data helps reduce the imbalance in predictions for each dataset separately and to include more spectra of different chemical types in the model.

As a result, Random Forest was chosen because of its slight performance advantage over XGBoost, and the combined set was chosen as the dataset. Bin size was chosen as 0.1, and MS peaks in each bin were represented as values of peak intensities in the corresponding *m*/*z* intervals, after evaluating different binning and peak representation strategies (see the Supporting Information).

### Model Performance

**Figure 2:**
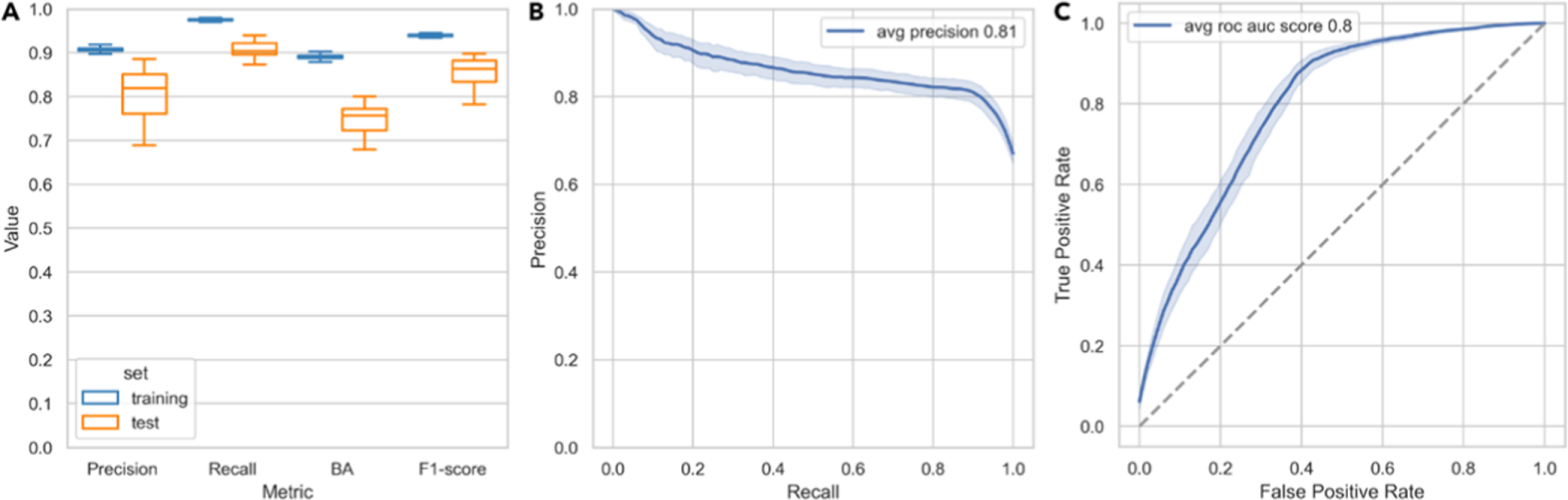
Performance of BitterMasS at 20 different splits: (A) Distribution of four metrics measured on training and test sets; (B) average precision–recall curves; and (C) average ROC curves. Bootstrap 95% confidence intervals for the mean are shown.

To test BitterMasS model effectiveness, we conducted a random splitting process on the training and test sets 20 times on the combined dataset. Random Forest models with preoptimized hyperparameters were trained, and their performance was tested. As a result, we obtained the distribution of the scores on the four metrics (Figure 2A) and plotted the average precision–recall curve (Figure 2B) and the average ROC curve (Figure 2C). The mean values of the metrics for the training and test sets are presented in Table 3.

**Table 3:** Mean Values of Metrics and Their Standard Deviation for 20 Splits, Train and Test Model

set	precision	recall	BA	F1-score
training	0.91 ± 0.01	0.98 ± 0.01	0.89 ± 0.01	0.94 ± 0.01
internal test	0.81 ± 0.05	0.91 ± 0.02	0.75 ± 0.03	0.85 ± 0.03

All models have similar performance on the training set regardless of metrics. When validated on the test set, there is a wider scattering of metric values. Since all models had close performances on the training set, the best model with the highest balanced accuracy on the test set was selected and used further.

The values of the naive model metrics on the training and test sets were as follows: precision = 0.67, recall = 0.67, BA = 0.51, and F1-score = 0.67. Thus, BitterMasS significantly outperformed the naive model with respect to all measured metrics.

### Feature Importance of the BitterMasS Model

**Figure 3:**
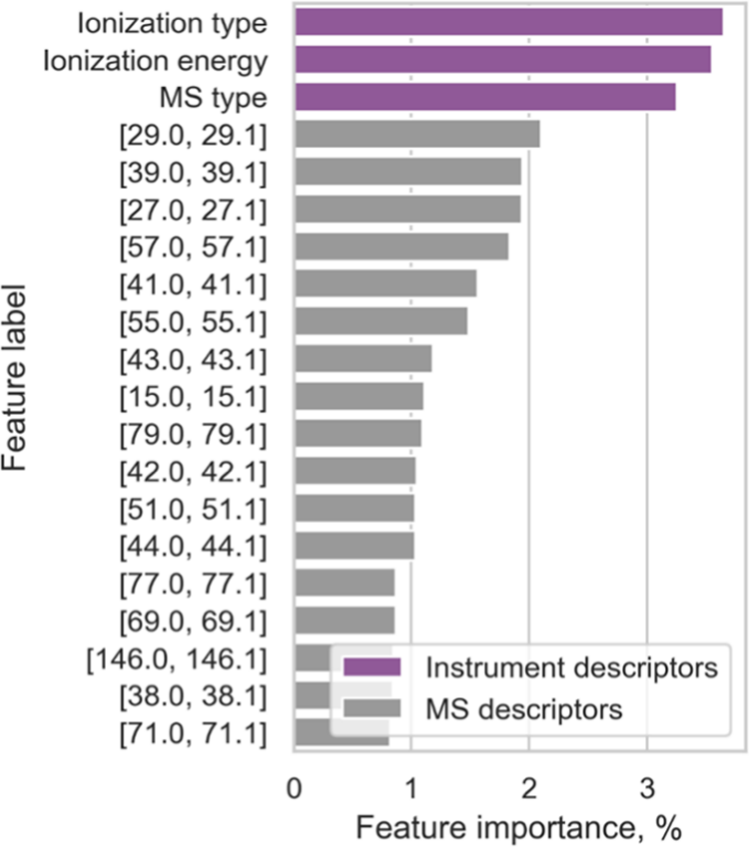
20 most important features for the BitterMasS model. The limits of *m*/*z* values for important bins are given in square brackets.

To obtain intuitive insights into the BitterMasS model, feature importance was extracted. In a Random Forest algorithm, on which the BitterMasS model is based, feature importance refers to the relative importance of each feature when making predictions. The feature importance values are relative and should be interpreted in the context of the other features in the model. Since the total importance of all features is equal to one, the values of each feature were expressed as a percentage and the 20 most important features are presented in Figure 3.

Three descriptors related to the measurement instrument were the most important ones. However, their total importance sums to only 10%, with the remaining 90% of the important features distributed over various bins of mass spectral descriptors.

**Figure 4:**
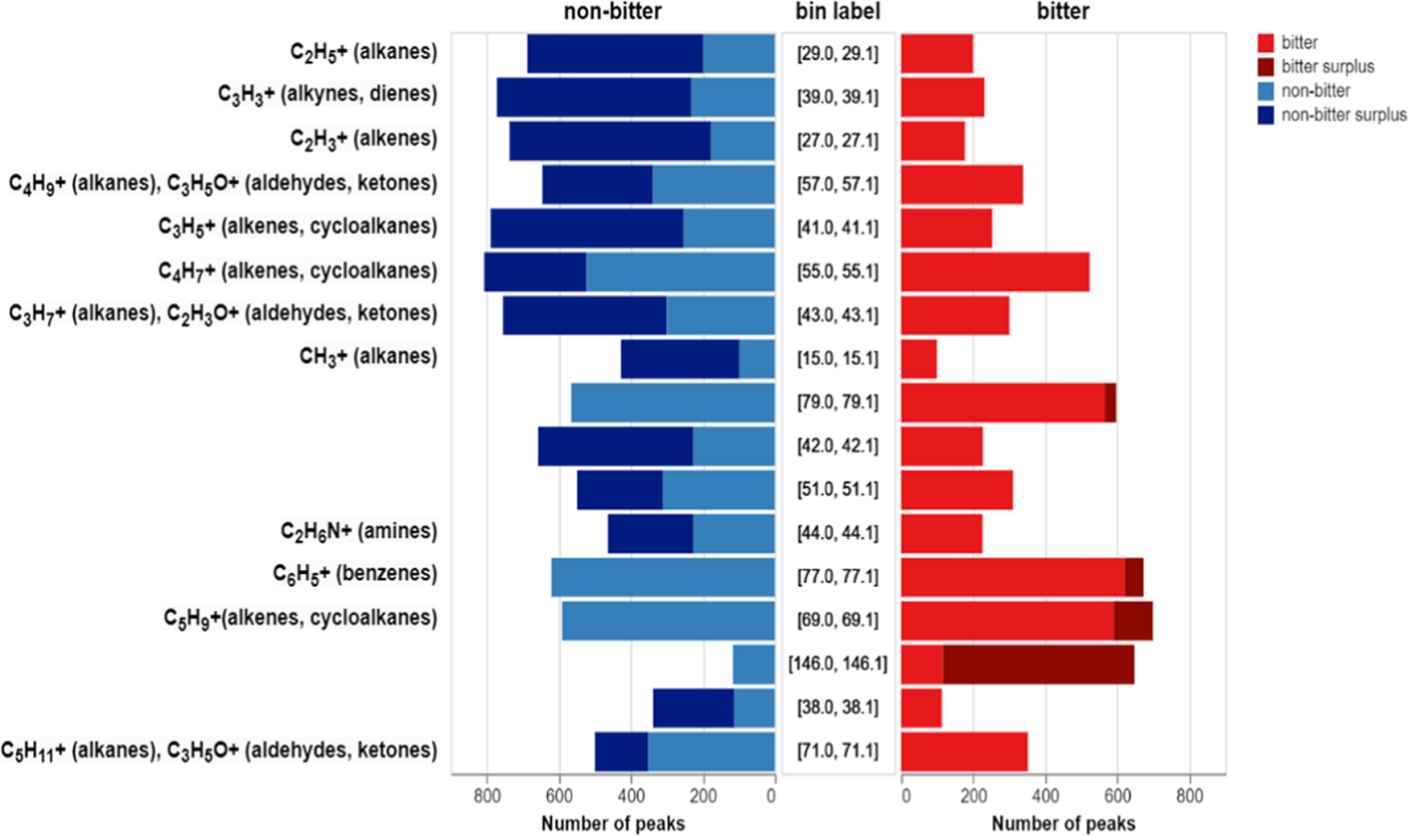
Popularity of peaks from bitter (red) and nonbitter (blue) mass spectra used for BitterMasS training. Darker colors show a surplus of the corresponding class.

Next, an attempt was made to link important bins to cation structures (Table S5). The number of peaks falling into 17 most important bins was counted (Figure 4) in bitter and nonbitter spectra. For some bins, there was a greater popularity among the nonbitter spectra, while for others, there was a greater popularity among the bitter spectra.

As can be seen from Figure 4, most of the important bins belong to a range of small values (up to 100 *m*/*z*). The fragments fitting these values are not specific to any particular class and are characteristic of all organic compounds. It is therefore difficult to say that any particular fragment is responsible for the bitterness of a compound. The excess of “nonbitter” fragments in the region up to 50 *m*/*z* can be explained by the greater popularity of small molecules among this class, which generate predominantly small fragments.

An interesting case is the bin with a range of *m*/*z* values from 146.0 to 146.1. It is populated frequently in spectra of bitter compounds, due to many different substructures of ions fitting this range.

### Predicting the Bitterness of the External Test Sets

In order to test performance, we collected new data from the literature to create an external test set (external set 1) and measured spectra for several additional compounds (external set 2).

**Figure 5:**
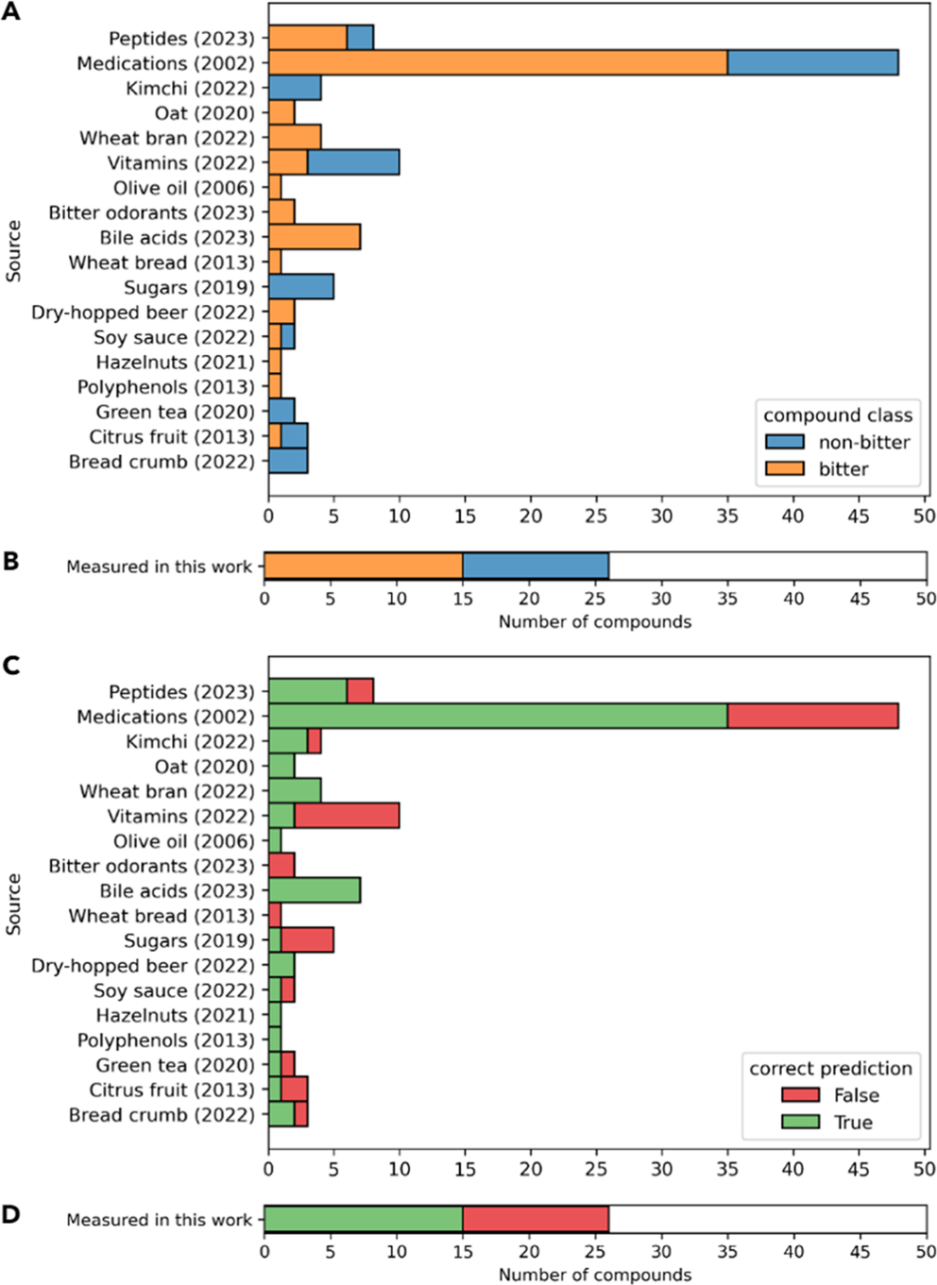
Distribution of bitter and nonbitter compounds from publications used for external set 1 (A) and external set 2 (B) and distribution of correct predictions by the BitterMasS model for compounds in the external set 1 (C) and external set 2 (D).

Prediction was performed as follows: Mass spectra were selected for each compound, and prediction was performed for each spectrum. The results were then combined, and the final label was selected based on the majority of the predictions. In case the numbers of “bitter” and “nonbitter” labels were equal, random selection was used. The prediction results on the external set 1 and the external set 2 for the BitterMasS models are shown in Table 4. Additionally, Figure 5 shows the distributions of bitter and nonbitter compounds from each source for external set 1 as well as the distributions of correct predictions of the BitterMasS model for these sources.

**Table 4:** Performance of BitterMasS and BitterPredict on the External Sets

model	no. of compounds	no. of mass spectra	precision	recall	BA	F1-score
External Set 1						
BitterMasS	106	759	0.67	0.93	0.57	0.78
BitterPredict	90		0.81	0.81	0.73	0.81
External Set 2						
BitterMasS	26	111	0.58	0.99	0.50	0.73
BitterPredict	11		0.50	0.99	0.58	0.67

To compare BitterMasS with other tools that predict bitterness, when the structure is known, we used BitterPredict,^14^ which predicts the bitterness of a chemical compound based on features calculated from its chemical structure.

Prediction was performed for compounds extracted from external test set 1 and external set 2 (Table 4) In each set, some of the compounds were not in the applicability domain^72^ defined for BitterPredict,^14^ so the model was not applicable (16 compounds were out for external set 1 and 15 compounds were out for external set 2). The BitterPredict applicability domain was defined as −3 ≤ *A* log *P* ≤ 7 and MW ≤ 700. It includes 97% of the bitter compounds used in the construction of BitterPredict.

The impact of training set size on the model’s predictive ability was further investigated, showing that increasing the training dataset size first dramatically and then moderately improved the model’s predictions (Figure S6).

Compared to the validation on the internal test set, BitterMasS has lower values for all computed metrics on both external sets. However, this efficiency remains acceptable for new data (precision = 0.67, recall = 0.93).

It can be concluded that for BitterMasS and BitterPredict, external set 2 is a greater challenge than external set 1. For both external sets, BitterPredict makes predictions more accurately than BitterMasS but with some reduction in the number of predictions due to the applicability domain restriction of BitterPredict.

The distribution of correct BitterMasS predictions for external set 1 as a function of the source shows that the model has difficulty predicting for nonbitter compounds, as indicated by the high recall values but low (close to 0.5) BA. However, for sources such as kimchi (2022)^53^ and bread crumbs (2022),^68^ the spectra for the nonbitter compound were classified correctly.

The results indicate that BitterPredict has a better performance, balanced accuracy, and F1-score than BitterMasS. This means that whenever the chemical structure of a compound is known, it is better to use BitterPredict than BitterMasS. However, the real question for BitterMasS is which is the preferable scenario when starting from the mass spectrum only: is it better to predict bitterness from the mass spectrum (as BitterMasS does) or to start from the spectrum, assign a structure, and then predict bitterness based on the assigned structure (i.e., using BitterPredict)? These scenarios are evaluated next.

### Testing “Spectrum–Bitterness” and “Spectrum–Structure–Bitterness” Strategies

The external sets 1 and 2 were merged and used to test the spectrum–bitterness vs spectrum–structure–bitterness strategies. Since mass spectra for the two nonbitter compounds in external set 2, l-pyroglutamic acid and adenosine monophosphate, were already found in the literature (external set 1),^53^^56^ the new spectra were added to them.

One mass spectrum for each compound in the external set (130 spectra) was randomly selected as a set of mass spectra for “unknown” compounds. In the spectrum–bitterness strategy, bitterness was predicted for each spectrum by using BitterMasS.

For the spectrum–structure–property strategy, the assignment of each of the 130 spectra was carried out by searching MassBank.^46^ Notably, for real-world scenarios, only a low percentage (5–10%)^32,33^ of identified compounds are expected. Next, bitterness was predicted for the annotated structures using BitterPredict, with 102 molecules (∼80% of those identified) falling into the applicability domain. For 73 compounds, BitterPredict predicted bitterness labels correctly. The results for both strategies to determine bitterness from the mass spectra of compounds are presented in Table 5.

**Table 5:** Test Results of Spectrum–Property and Spectrum–Structure–Property Strategies on External Set 1

strategy	no. of compounds	precision	recall	BA	F1-score
spectrum–bitterness (BitterMasS)	130	0.66	0.93	0.55	0.77
spectrum–structure–bitterness (BitterPredict)	102	0.65	0.65	0.53	0.65

In a real-life scenario (i.e., a single mass spectrum per compound, with only some assignment of the spectrum to the structure is possible), the direct spectrum–bitterness approach using BitterMasS is clearly a much better option. This is mainly because predictions can be provided for all spectra, while going through structural assignment prevents predictions for many of the spectra, and the applicability is narrowed even further due to the BitterPredict applicability domain restrictions.

## Discussion

BitterMasS was developed to predict the bitterness of a compound based on its mass spectrum. In the current implementation, the model makes predictions based on EI-MS or ESI-MS/MS spectra obtained in positive ion mode.

Various sources were combined to obtain a dataset of 2,511 compounds, comprising 1418 bitter and 1579 nonbitter compounds. Experimental EI-MS and ESI-MS/MS positive ion mode mass spectra for these compounds were extracted from the MassBank database,^46^ resulting in at least one spectrum for 30% of the molecules in the dataset. Prediction on the individual EI-MS and ESI-MS/MS sets showed poor results compared with the combined set.

The analysis of the importance of features for the model showed that instrumental descriptors reporting the type of MS, ionization method, and ionization energy at which the mass spectrum was obtained were the most important descriptors. The remaining 90% of the importance derives from accumulation of multiple individual peak bins, that is, the model uses meta information about the spectrum but predominantly uses information from multiple individual peaks for prediction.

To further validate the BitterMasS model, an external set was assembled based on 18 publications with sensory studies^51−52,53,60,61,68^ and laboratory measurements performed as part of this work.

The results of testing BitterMasS on the external set showed some imbalance in terms of accuracy in predicting bitter and nonbitter spectra: precision = 0.67 and recall = 0.93 (external set 1) and precision = 0.58 and recall = 0.99 (external set 2). The high recall with low precision shows that the model tends to overpredict bitterness. We assume that diversifying the mass spectra of nonbitter compounds will provide improved performance in future versions of BitterMasS.

BitterPredict^14^ is a tool that predicts the bitterness of a compound based on its chemical structure. Physicochemical descriptors are used as inputs to the model. BitterMasS is a tool that predicts the bitterness of a compound based on its mass spectrum.

When both mass spectra and chemical structures of the compounds are known, BitterPredict (precision = 0.81 and recall = 0.81) outperformed BitterMasS (precision = 0.67 and recall = 0.93) in precision but had a slightly lower recall. For external set 2, both tools had high recall, but BitterMasS (precision = 0.58) had a slightly higher precision than that of BitterPredict (precision = 0.50). However, higher values for the BA and F1-score metrics were observed for BitterPredict compared to BitterMasS for both external sets, suggesting that when the chemical structure is available, BitterPredict is a better strategy.

We next checked which tool is better at predicting the bitterness of a compound using the mass spectrum data only. This scenario simulates real-life metabolomic analyses aimed at investigating the composition of a product or a sample. In such cases, mass spectral information is more readily available.

We compared BitterMasS direct prediction from mass spectra to the spectrum–structure prediction strategy, where there are three stages, each with potential efficiency loss: annotating the structure from the spectrum (which is the most problematic stage), passing the applicability domain of BitterPredict, and finally the prediction itself. Although precision and balanced accuracy were similar (0.65–0.66 for precision and 0.53–0.55 for balanced accuracy), recall and the F1-score were higher when using direct prediction of the bitterness spectrum using BitterMasS (0.93 vs 0.65 for recall and 0.77 vs 0.65 for the F1-score). Therefore, the direct prediction of bitterness from the mass spectrum is recommended when the chemical structure of a compound is not yet unknown.

It is important to note that only 30% of the compounds in the initial bitter and nonbitter sets in the training set had at least one mass spectrum identified. Most of the articles reporting experimental mass spectra do not imply the actual spectra. Standardized mass spectral deposition will help train the next generation of BitterMasS and other spectrum–property approaches.

We developed the BitterMasS tool to predict the bitterness of a compound based on its EI-MS or ESI-MS/MS spectrum. Mass spectrometry is insensitive to chirality, which, in turn, limits BitterMasS. Indeed, two taste molecules in the external set that were misclassified by BitterMasS are enantiomers and have identical mass spectra. In the future, this approach can be extended to include other measurements, including circular dichroism (CD), spectrophotometric measurements, and more.

Analysis of impact of data size on the performance of BitterMasS suggested that improvement can be expected by significantly increasing the nonbitter compound spectral representation in the training set.

The good performance of BitterMasS suggests applications in food and agriculture. For example, mass spectra from multiple cultivars, samples, treatments, or fractions can be analyzed to predict bitterness. Furthermore, BitterMasS is a unique tool for “illuminating” the bitter-tasting part of the “dark” metabolome.

## Supporting Information Available

Additional data used in the development of BitterMasS, chemical structures, and corresponding mass spectra used in external set 2 (PDF)
