# Supplementary material for: BitterMasS: Predicting Bitterness from Mass Spectra
Source: J Agric Food Chem. 2024 Apr 30;72(18):10537–47. doi: 10.1021/acs.jafc.3c09767 (PMC11082931; doi:10.1021/acs.jafc.3c09767)
Supplement: Supplementary file 1 — jf3c09767_si_001.pdf [file jf3c09767_si_001.pdf]

BitterMasS: predicting bitterness from mass spectra

Evgenii Ziaikin[1], Edisson Tello[2], Devin G. Peterson[2] and Masha Y. Niv[1*]

[1]Food Science and Nutrition, The Robert H Smith Faculty of Agriculture, Food and Environment, The Institute of Biochemistry, Food and Nutrition, The Hebrew University of Jerusalem, 76100 Rehovot, Israel

[2]Department of Food Science and Technology, College of Food, Agriculture, and Environmental Sciences, The Ohio State University, 43210 Ohio, USA

*correspondence to masha.niv@mail.huji.ac.il

Supplementary materials used in the development of BitterMasS

Expanding the dataset:

Table S1. Number of mass spectra for bitter and non-bitter compounds in prepared datasets extracted from MassBank of North America (MoNA) (1). The number of new compounds or spectra discovered in MoNA, compared to those found in MassBank (2), is presented in parentheses.

**Dataset**	**# of compounds (# of additional compounds)**		**# of spectra (# of additional spectra)**	
	**Bitter**	**non-bitter**	**bitter**	**non-bitter**
**EI-MS**	204 (31)	499 (5)	492 (79)	1,178 (58)
**ESI-MS/MS**	266 (22)	92 (24)	3,347 (183)	1,000 (283)
**Combined dataset**	374 (26)	538 (26)	3,839 (262)	2,178 (341)

Impact of multiple spectra of the same molecule:

To illustrate that different spectra of the same compound can be similar or dissimilar, the compound with the highest number of available spectra in the set was chosen. This was the bitter compound Antipurine (Figure S1A), which has 156 spectra (2 EI-MS and 154 ESI-MS^2). It is an analgesic and antipyretic that is administered by mouth and as ear drops. The similarity between the spectra was calculated using cosine similarity (equation 1). This is a popular metric to compare and contrast mass spectra. The tolerance value at which the m/z values were considered to be the same was 0.1.

$$\text{cosine similarity} = \frac{AB}{||A||\,||B||}(1)$$

Unique pairwise cosine similarity values were calculated for 154 Antipurine ESI-MS^2 spectra and the resulting distribution is shown in Figure S1B.

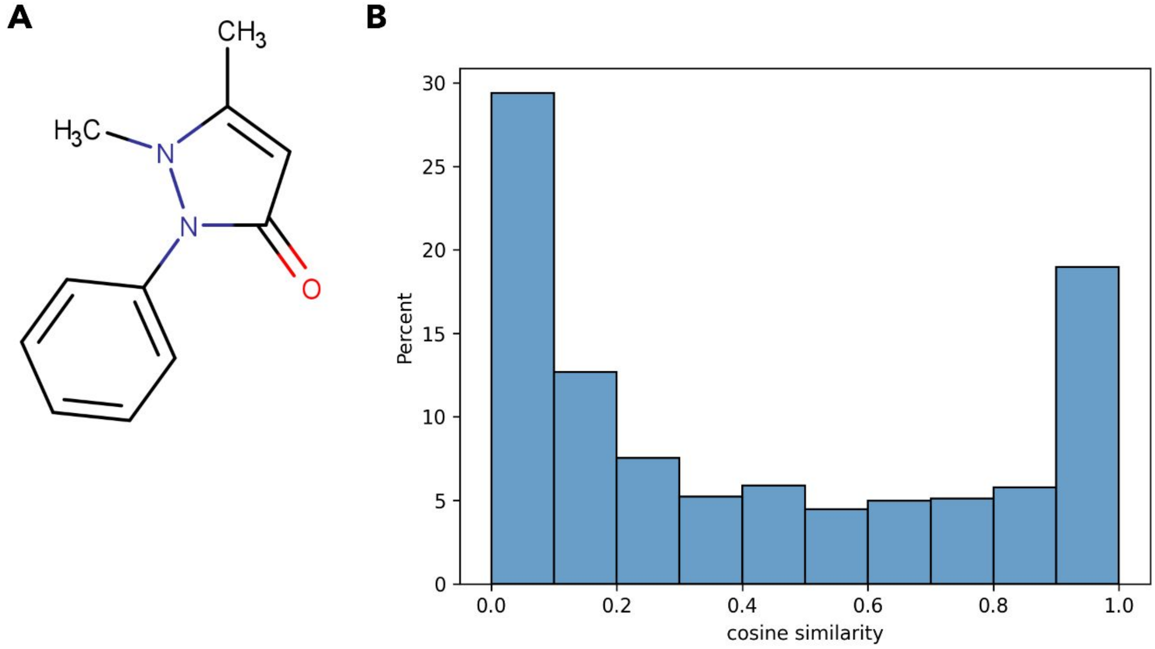

Figure S1. (A) The chemical structure of Antipurine, (B) Pairwise similarities of mass spectra found in MassBank for Antipurine.

As an example of similar and dissimilar spectra, Figure S2 shows a pairwise comparison of Antipurine spectra with different values of cosine similarity. In the comparison of similar spectra (Figure S2A) the main peaks with large intensities coincide. In the other case (Figure S2B) the main peaks are shifted toward lower m/z values. Although the spectra were obtained for the same compound and the same types of instruments were used (ESI-MS2), these spectra are very different. This can be explained by different ionization energies. In the case of higher energies, the original molecule breaks up into smaller fragments, and, accordingly, its baseline peak will lie in the region of low m/z.

Based on the resulting similarity distribution of Antipurine spectra, only about 20% of the spectra had values between 0.9 and 1.0, i.e. very similar spectra. The remaining values between 0.3 and 0.9 were evenly distributed (about 5% for each bin). The surprising finding was that a large group of similarity values between 0 and 0.1 (about 30%), i.e. highly dissimilar spectra. This further confirms that a variety of different mass spectra

sources, capture modes, ionisation energies etc. generate a multitude of both similar and very different mass spectra even for the same compound.

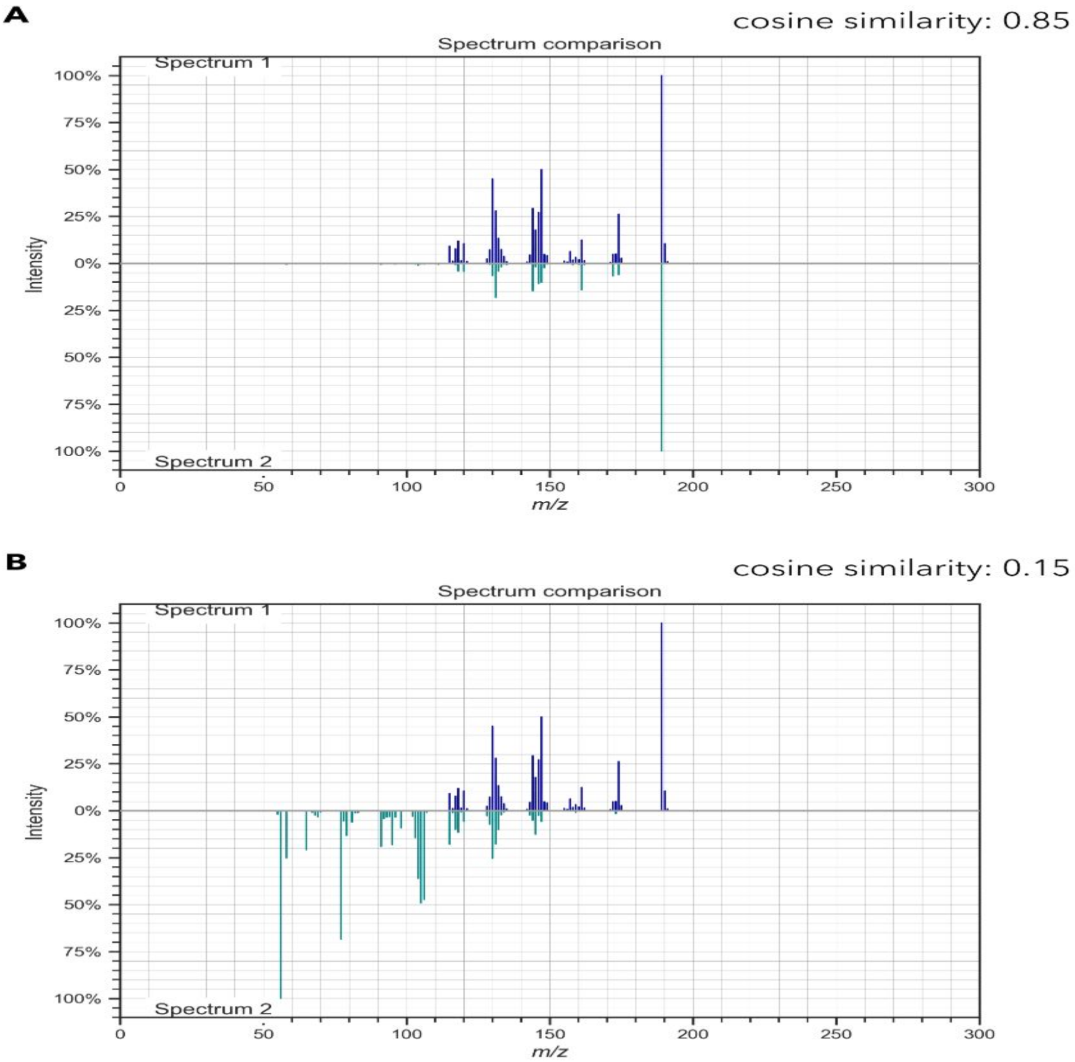

Figure S2. Pairwise comparisons of the ESI-MS2 spectra of Antipurine: (A) Similar spectra (cosine similarity = 0.85) and (B) Dissimilar spectra (cosine similarity = 0.15).

Model parameters:

Table S2. Names of parameters and their values used in Random Forest model optimization.

**Parameter**	**Values**
Number of trees	50, 100, 150, 200

Splitting criterion	gini, entropy
Max depth	10, 15, 20
Max features	sqrt, log2
Class weight	balanced, balanced subsample

Table S3. Names of parameters and their values used in XGBoost model optimization.

**Parameter**	**Values**
Number of estimators	20, 30, 40, 50
Learning rate	0.01, 0.1
Max depth	3, 4, 5
Subsample	0.5, 0.75. 1.0

Table S4. Training and validation results of Random Forest and XGBoost models on the EI-MS, ESI-MS^2 and combined datasets.

**Dataset**	**Training set**				**Test set**			
	**Precision**	**Recall**	**BA**	**F1 score**	**Precision**	**Recall**	**BA**	**F1 score**
**Random Forest**								
**EI-MS**	0.99	0.98	0.99	0.98	0.57	0.29	0.60	0.38
**ESI-MS^2**	0.99	0.80	0.90	0.89	0.85	0.63	0.52	0.72
**Combined**	0.91	0.98	0.90	0.94	**0.82**	**0.92**	**0.73**	**0.87**
**XGBoost**								
**EI-MS**	0.99	0.74	0.87	0.85	0.52	0.33	0.60	0.41
**ESI-MS^2**	0.88	0.99	0.71	0.93	0.85	0.99	0.52	0.92
**Combined**	0.89	0.94	0.86	0.92	**0.82**	**0.91**	**0.72**	**0.86**

For the EI-MS dataset, overfitting was observed for both Random Forest and XGBoost methods, even when applying 5-fold cross-validation in hyperparameter optimization, as manifested in significant drop of performance on test set compared to training set. In the case of ESI-MS^2 set, metrics such as Precision, Recall and F1-measure show high values on both training and test sets. However, the Balanced accuracy on the test set indicates that the model fails to make correct predictions for each class. This, and the high Recall, indicates that the model tends to overpredict "bitter". The combined dataset leads to the best results (shown in bold), including 0.72-0.73 balanced accuracy,

Selection of bin size

Peak descriptors can be generated with different bin sizes. The smaller the size value, the more detailed the descriptors will be, and vice versa. In order to determine which bin size is more effective four BitterMasS, models were built with bin sizes of 0.1, 0.2, 0.5 and 1.0. Values of size less than 0.1 were not used because vectors predominantly consisting of 0. Intensities were used as the type of peak descriptors.

According to the results obtained, all 4 models perform well on the training and test sets, Figure S3A. However, using lower values of bin size reduces the overfitting of the model as the difference between the metrics values on the training and test set decreases, Figure S3B. Thus, the bin size of 0.1 was chosen for training the final model.

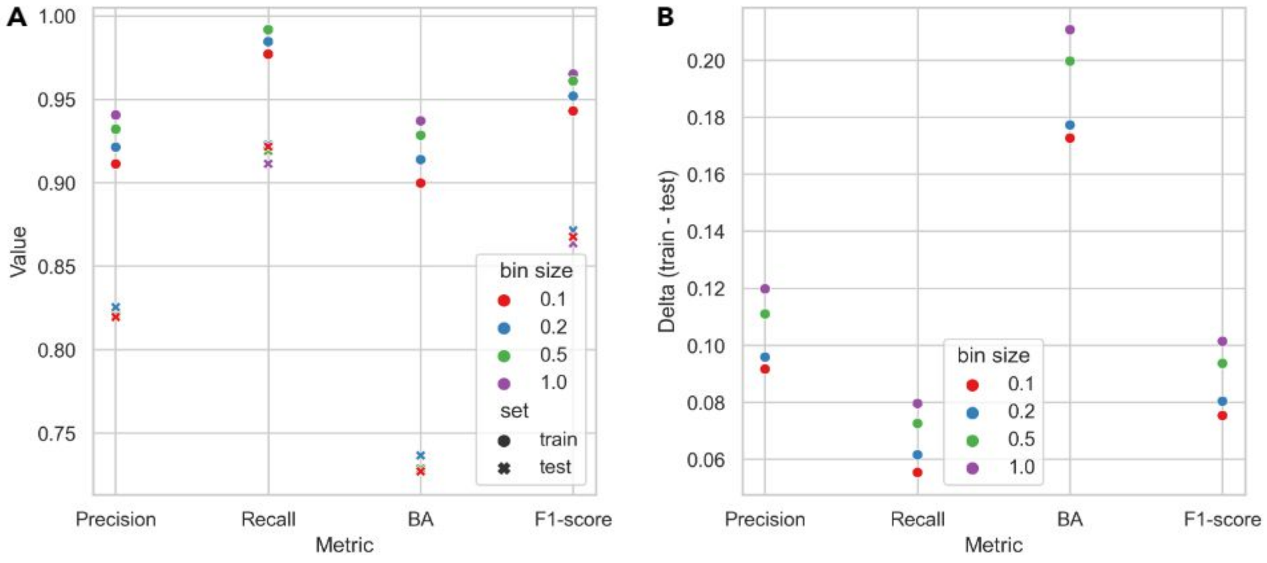

Figure S3. Performance results of BitterMasS models at different bin sizes in intensity-based peak descriptors: (A) Metrics values when validating models on training and test sets; (B) Difference between model performance values (training - test). The lower the difference, the less the model is overfitted on the training set.

Selection of peak descriptors type

Three strategies were used to generate peak descriptors: peak intensity, presence of peak in a bin and number of peaks in a bin. The bin size was 0.1. According to the results obtained, all three strategies show approximately the same performance on the training and test sets Figure S4A. However, the strategy using peak intensity has a slightly smaller difference between the performance on the training and test sets Figure S4B. It was chosen as the peak descriptor generation strategy in the final model.

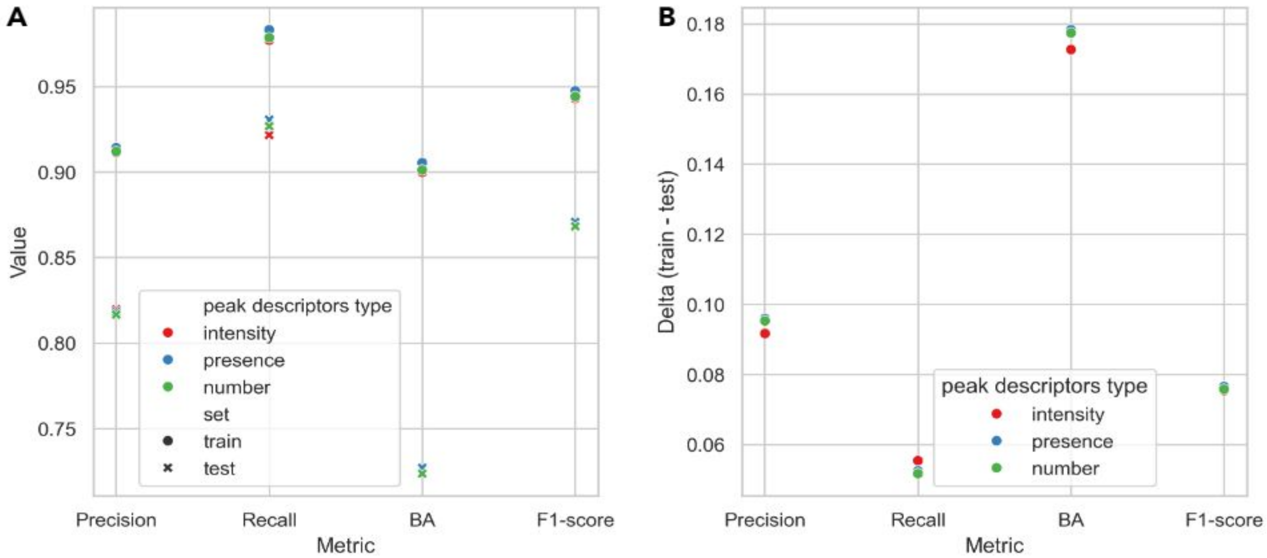

Figure S4. Performance results of BitterMasS models at different types of peak descriptors with bin size 0.1: (A) Metrics values when validating models on training and test sets; (B) Difference between model performance values (training - test). The lower the difference, the less the model is overfitted on the training set.

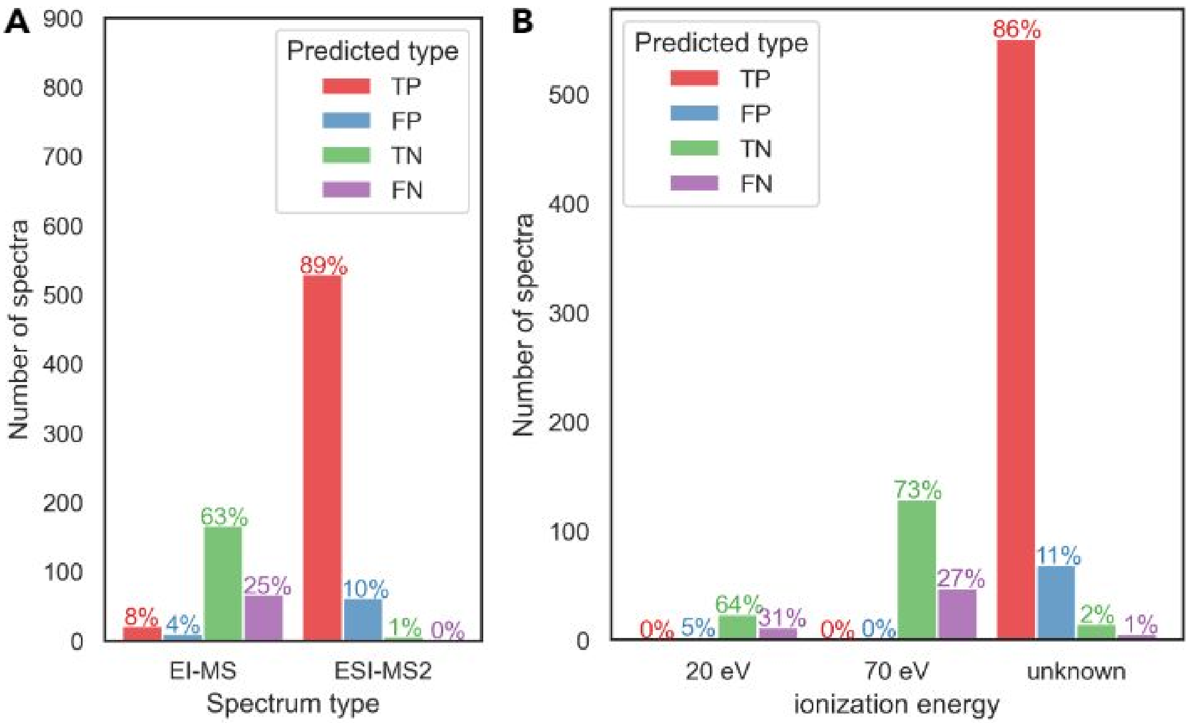

Figure S5. Error analysis on the test set by the BitterMasS model distributed by (A) Spectrum Type; (B) Ionization energy.

Table S5. 17 bin labels and ion formulas that fit the m/z values in descending order of importance to the model.

Bin label	Ion	Importance, %
**[29.0, 29.1]**	C_2H_5+ (alkanes)	2.1
**[39.0, 39.1]**	C_3H_3+ (alkynes, dienes)	1.9
**[27.0, 27.1]**	C_2H_3+ (alkenes)	1.9
**[57.0, 57.1]**	C_4H_9+ (alkanes), C_3H_5O+ (aldehydes, ketones)	1.8
**[41.0, 41.1]**	C_3H_5+ (alkenes, cycloalkanes)	1.6
**[55.0, 55.1]**	C_4H_7+ (alkenes, cycloalkanes)	1.5
**[43.0, 43.1]**	C_3H_7+ (alkanes), C_2H_3O+ (aldehydes, ketones)	1.2
**[15.0, 15.1]**	CH_3+ (alkanes)	1.1
**[79.0, 79.1]**	-	1.1
**[42.0, 42.1]**	-	1.0
**[51.0, 51.1]**	-	1.0
**[44.0, 44.1]**	C_2H_6N+ (amines)	1.0
**[77.0, 77.1]**	C_6H_5+ (benzenes)	0.9
**[69.0, 69.1]**	C_5H_9+(alkenes, cycloalkanes)	0.9
**[146.0, 146.1]**	-	0.8
**[38.0, 38.1]**	-	0.8
**[71.0, 71.1]**	C_5H_{11}+ (alkanes), C_3H_5O+ (aldehydes, ketones)	0.8

17 bin labels and ion formulas that fit the m/z values in descending order of importance to the model. For some bin labels it was not possible to identify the ion. In other cases, the number of suitable ion structures for the m/z value was so large that it was not possible to represent them as a specific gross formula or class. The larger the m/z value of an ion, the greater the number of structures that can be fit.

Effect of data size:

We performed 20 random splits into training and test sets at a ratio of 80% and 20%. Then 19 subsets were created based on each training set, first ranging from 1% to 10% in 1% increments (10 subsets), and then ranging from 20% to 100% in 10% increments (9 subsets). The BitterMasS model was trained on each subset and validated on the test set (Figure S6).

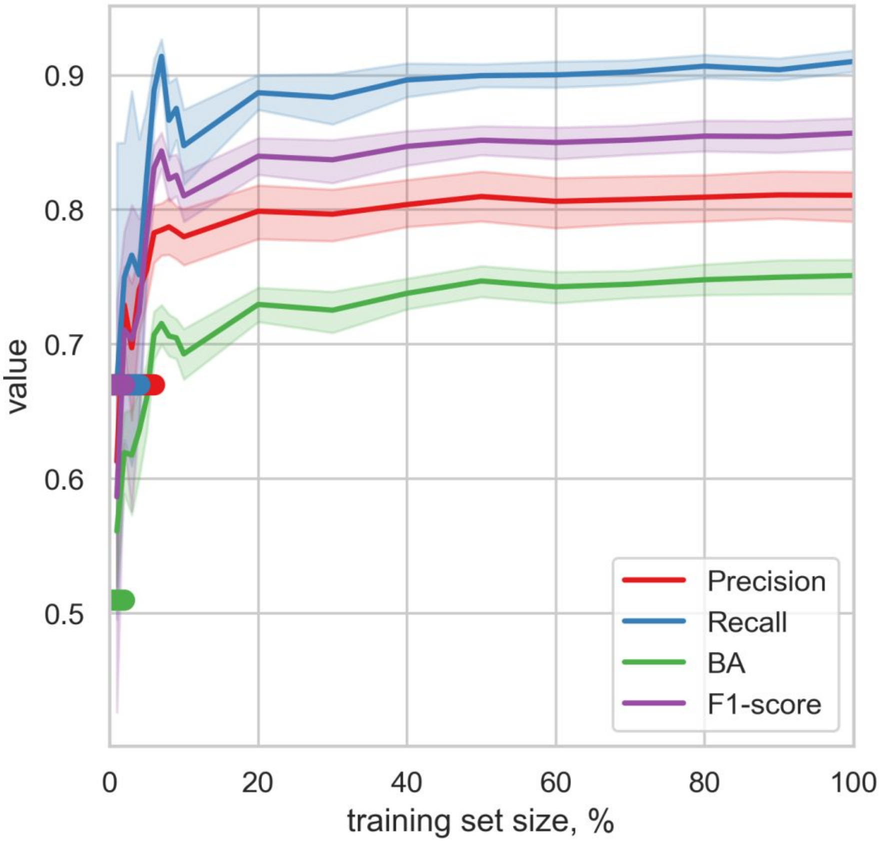

Figure S6. Results of the effect of training set size based on a 20-fold split of the initial set followed by an increase in the training set. Semicircles show the performance of the naive model metrics, which assumes for any spectra 66% chance of belonging to a bitter compound and 34% of belonging to a non-bitter compound, based on the bitter/non-bitter compounds composition of the training set.

The results show that when less than 10% of the data is used for training, there is a collapse with respect to all measured metrics, approaching a naive model that makes

predictions based on the proportion of bitter and non-bitter compounds in the training. Since the ratio of bitter to non-bitter class 66% to 34%. a naive model metric values are precision = 0.67, recall = 0.67, BA = 0.51, and F1-score = 0.67. (Figure S6).

The increase in model performance is dramatic moving from 10% to 20% of the data. The improvement of the performance is very moderate afterwards. Significant further improvement of the model in the future will require improving the size and diversity, mainly of the currently under-represented non-bitter set.

Structures of compounds and their mass spectra used as 2 external test set

a) *Cis*-isocohumulone alpha-acid

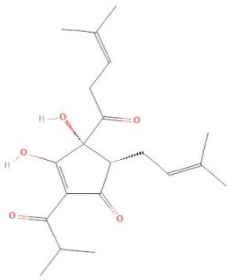

Collision Energy: 5eV

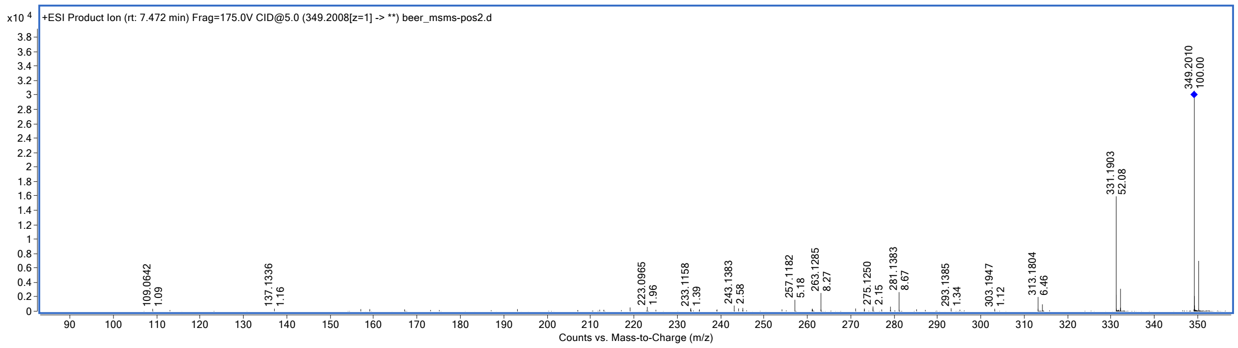

Collision Energy: 15eV

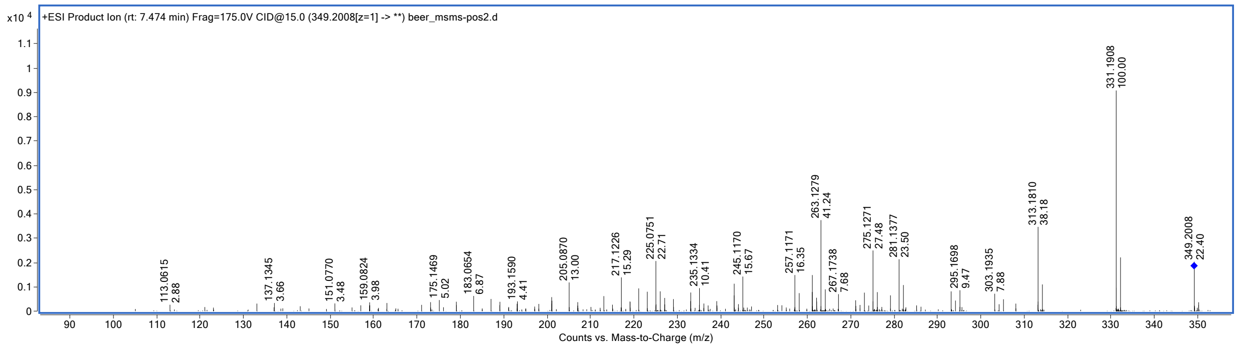

Collision Energy: 30eV

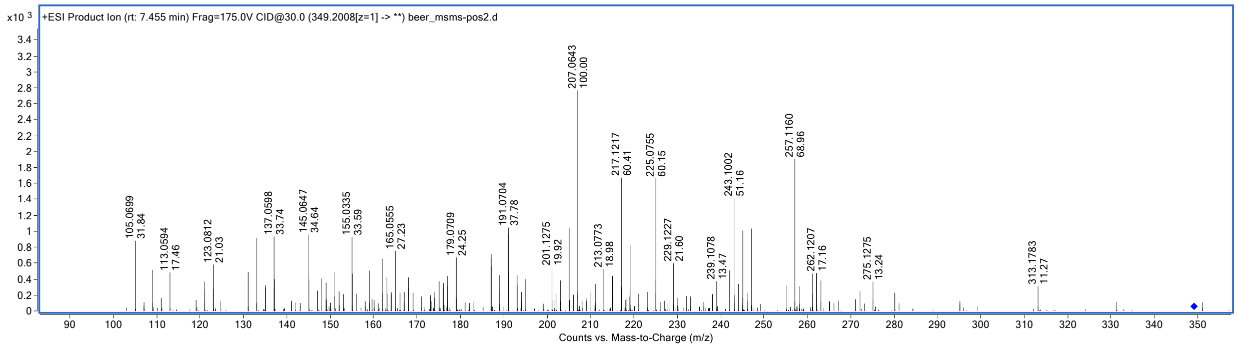

b) *Cis*-iso-N-Humulone

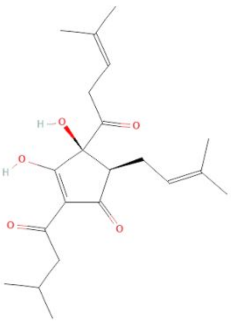

Collision Energy: 5eV

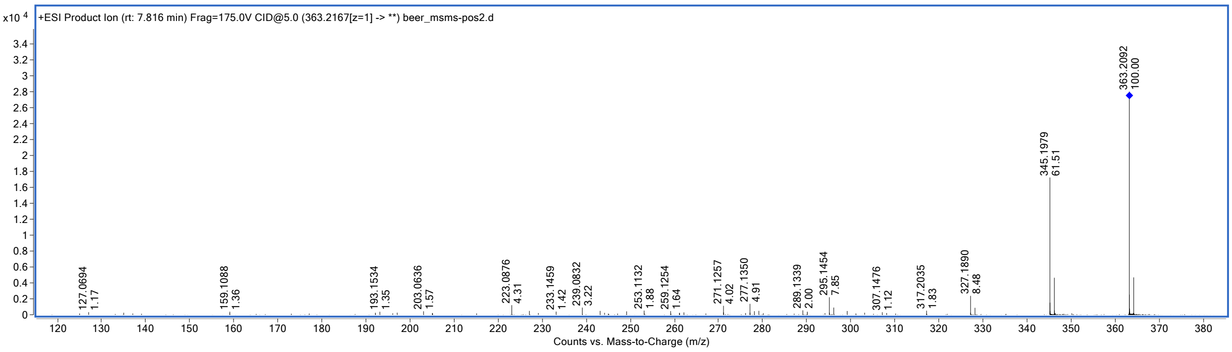

Collision Energy: 10eV

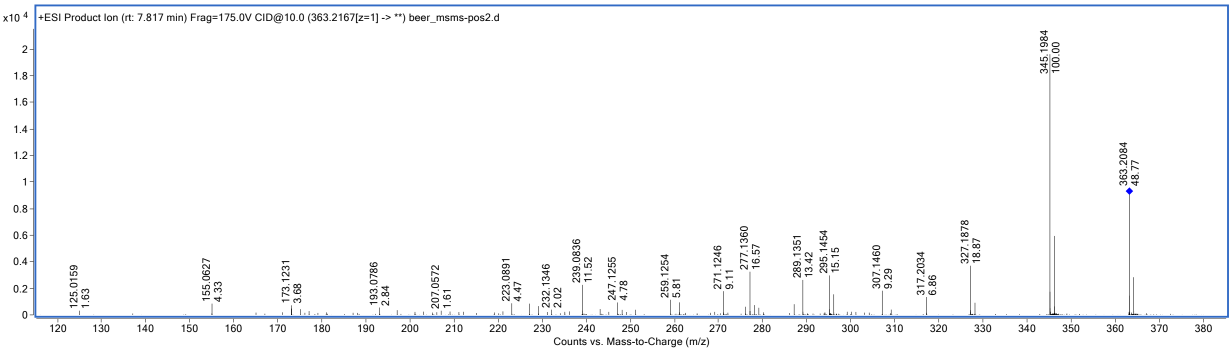

Collision Energy: 15eV

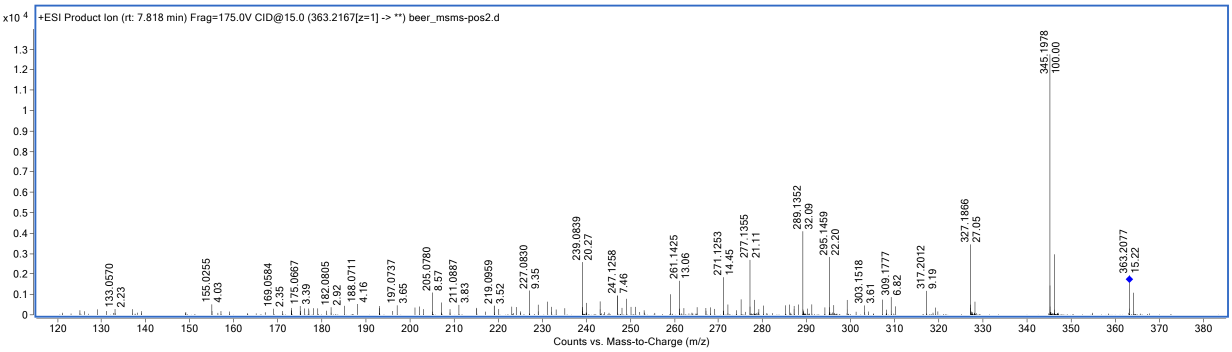

c) *Cis*-iso-adhumulone

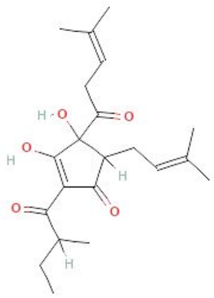

Collision Energy: 5eV

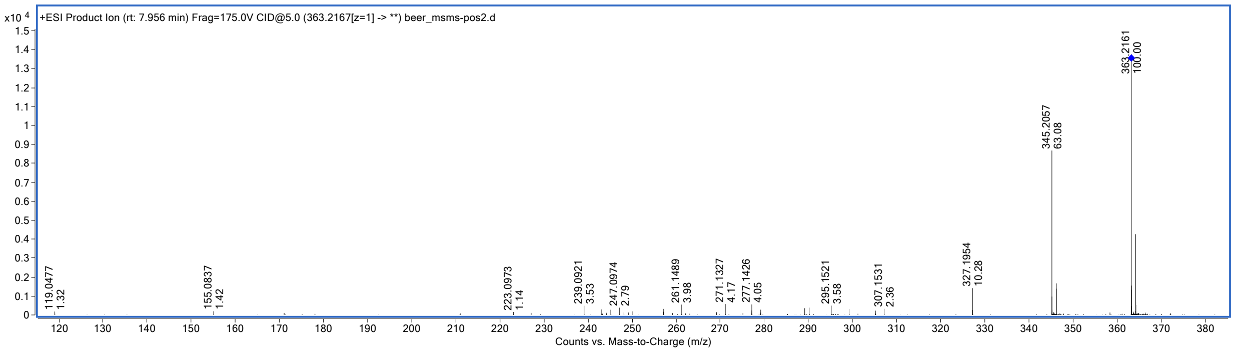

Collision Energy: 10eV

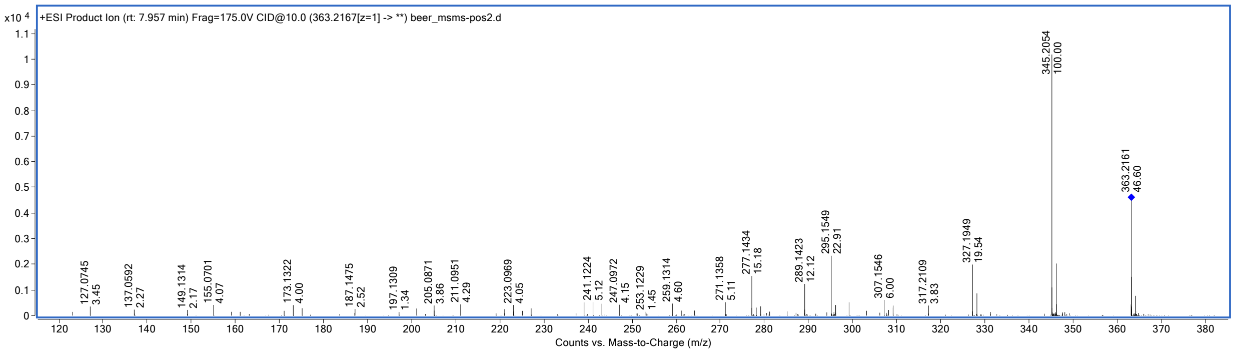

Collision Energy: 15eV

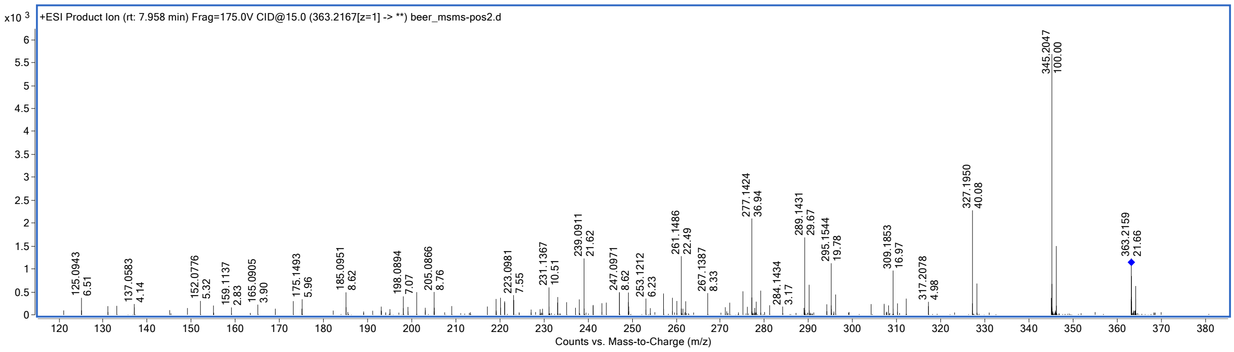

d) *alpha*-chaconine

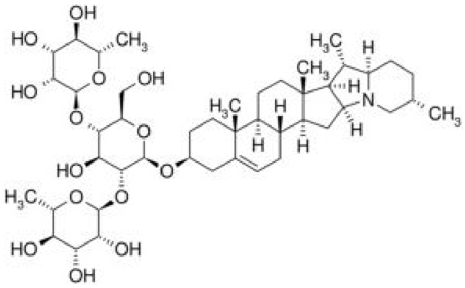

Collision Energy: 50eV

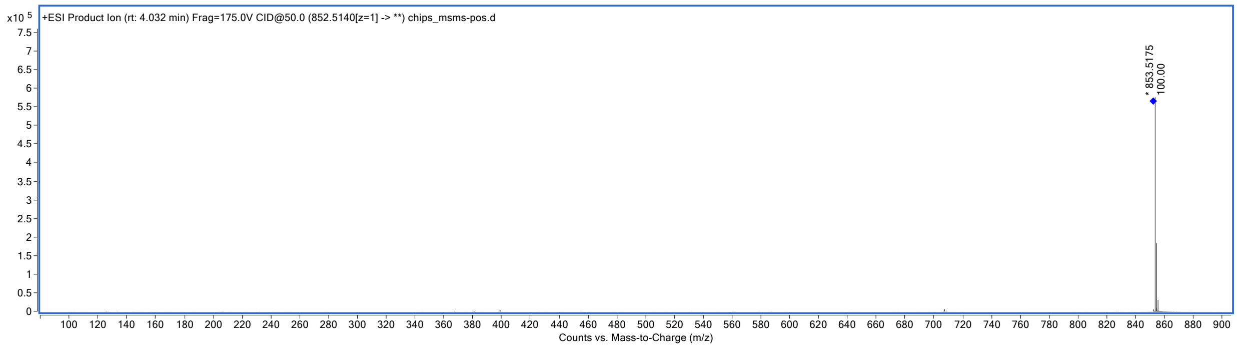

Collision Energy: 100eV

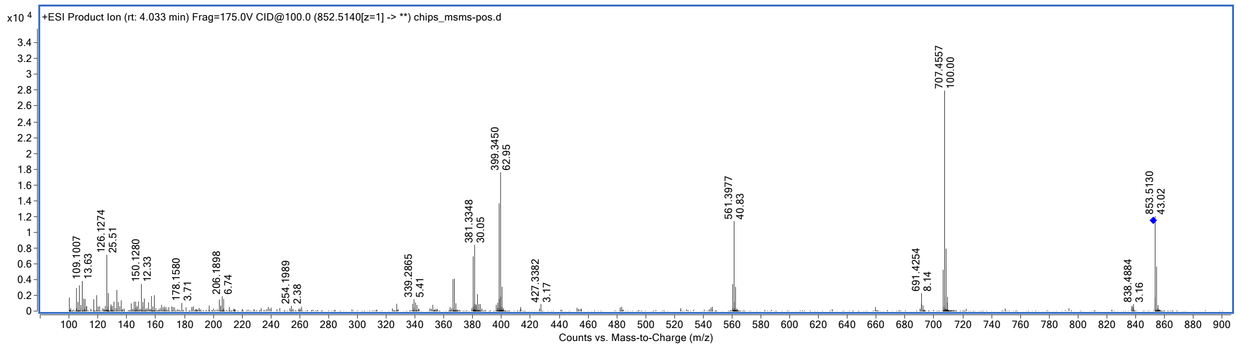

Collision Energy: 125eV

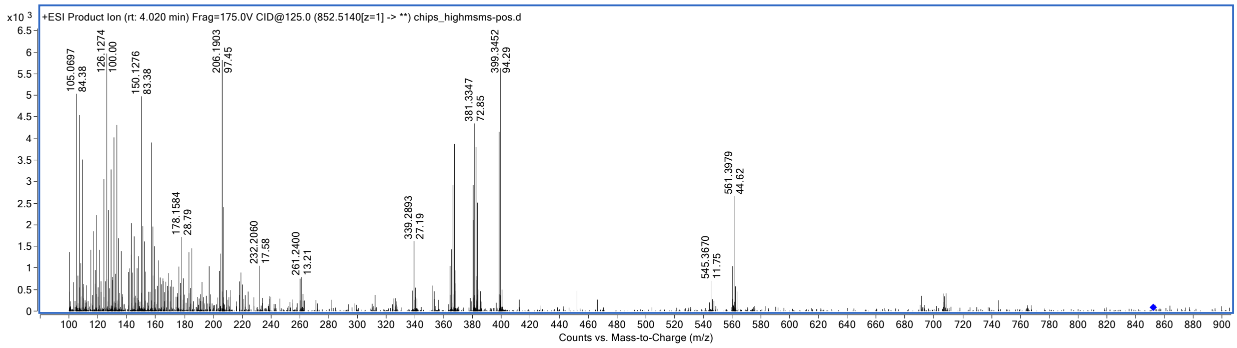

e) *alpha*-solanine

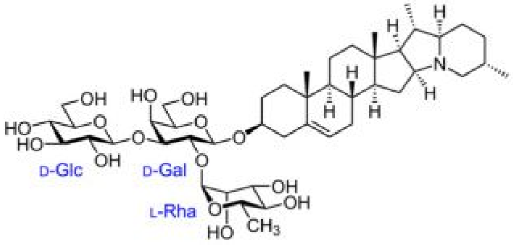

Collision Energy: 50eV

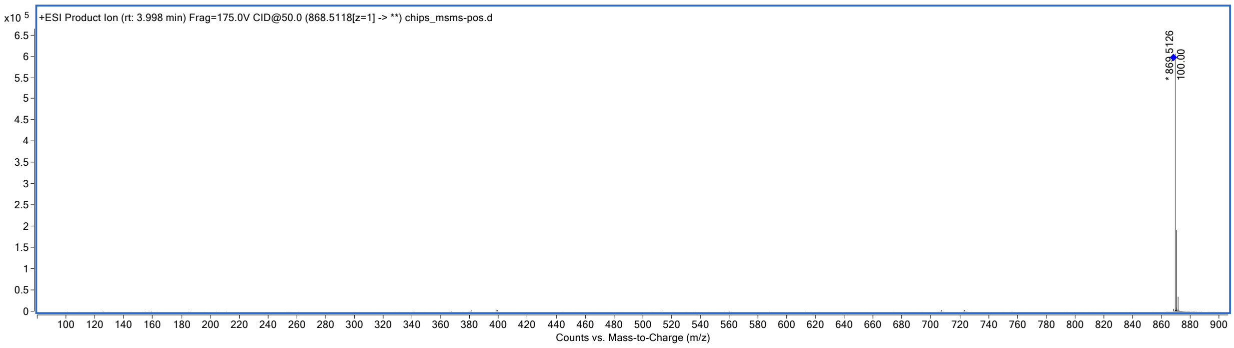

Collision Energy: 100eV

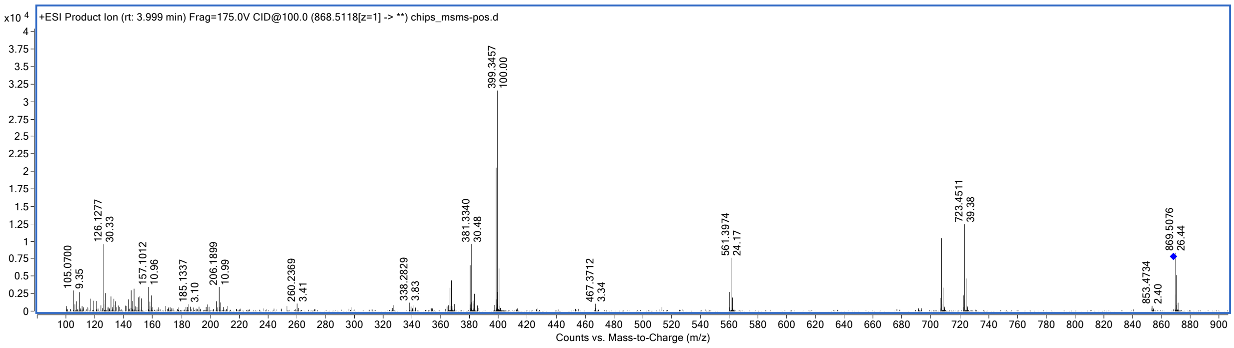

Collision Energy: 125eV

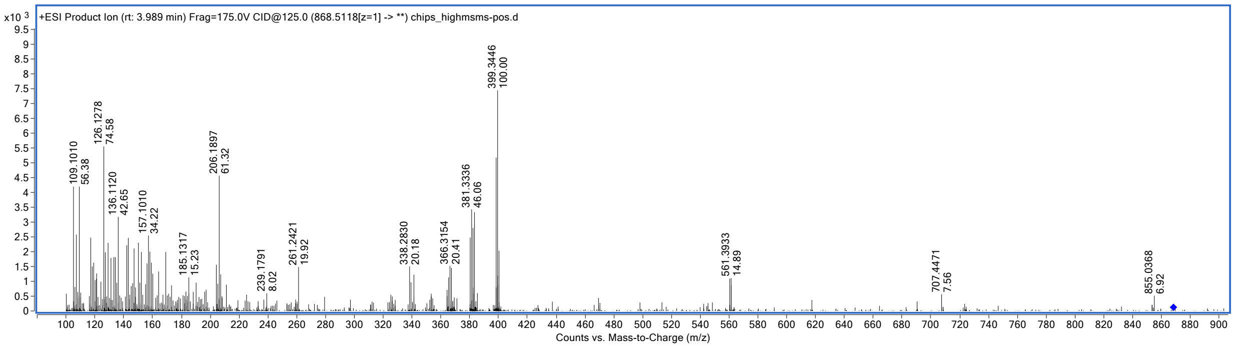

f) 3-O-Caffeoylquinic acid

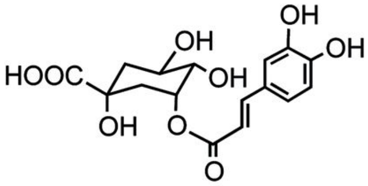

Collision Energy: 5eV

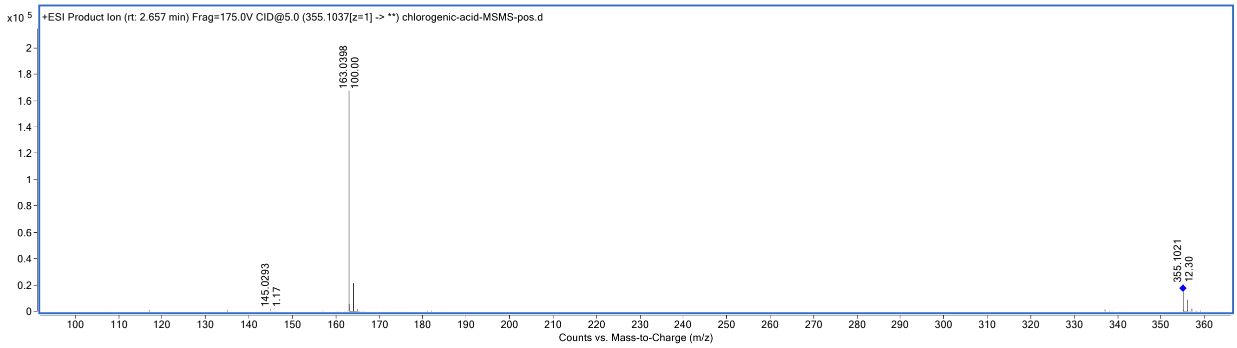

Collision Energy: 30eV

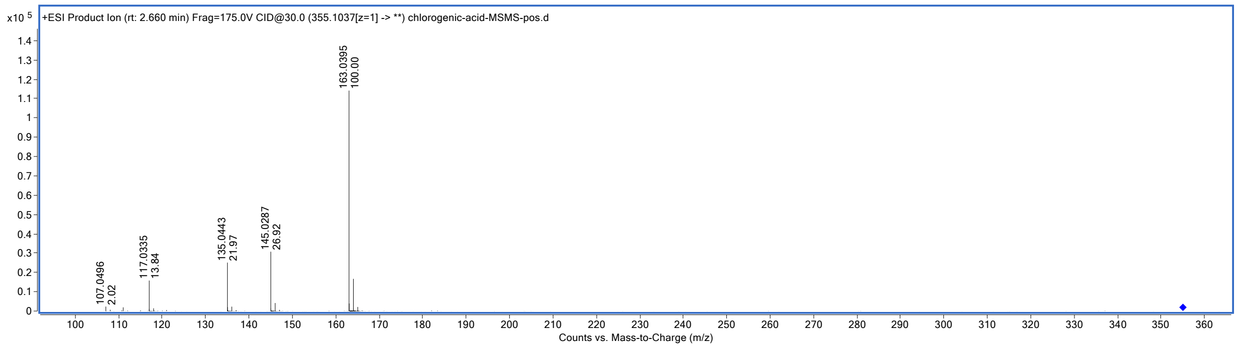

Collision Energy: 50eV

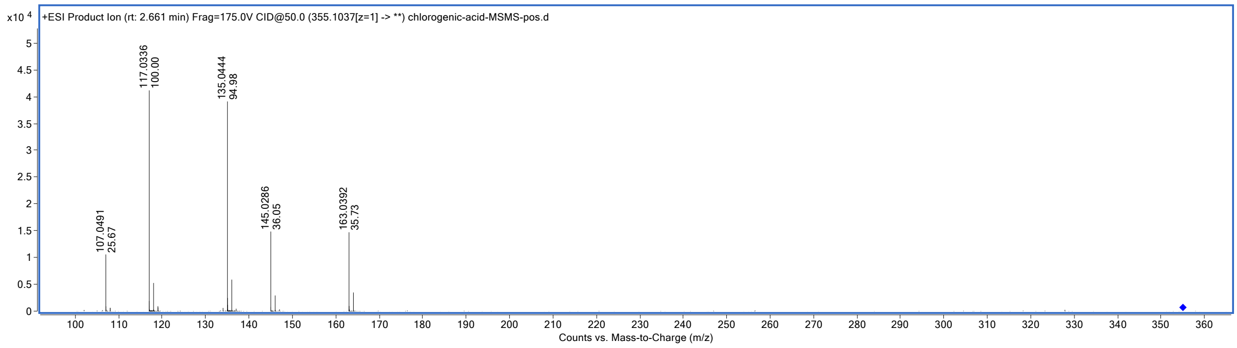

g) 2-(3-hydroxy-2-oxoindolin-3-l) acetic acid 3-O-6′- glucopyranosyl-2″-(2″oxoindolin-3″yl) acetate (Isomer 1)

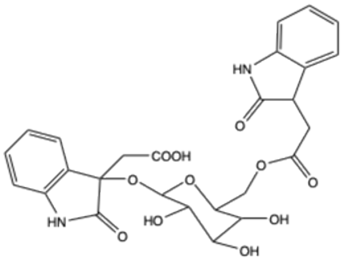

Collision Energy: 5eV

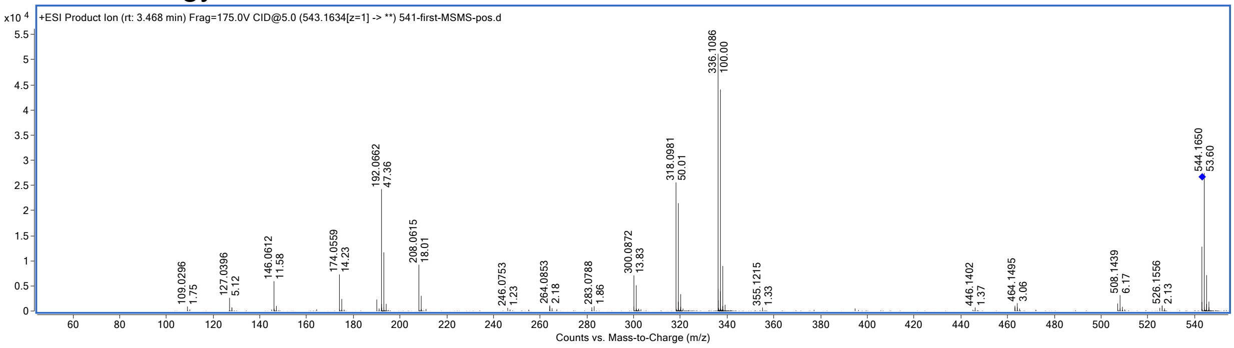

Collision Energy: 10eV

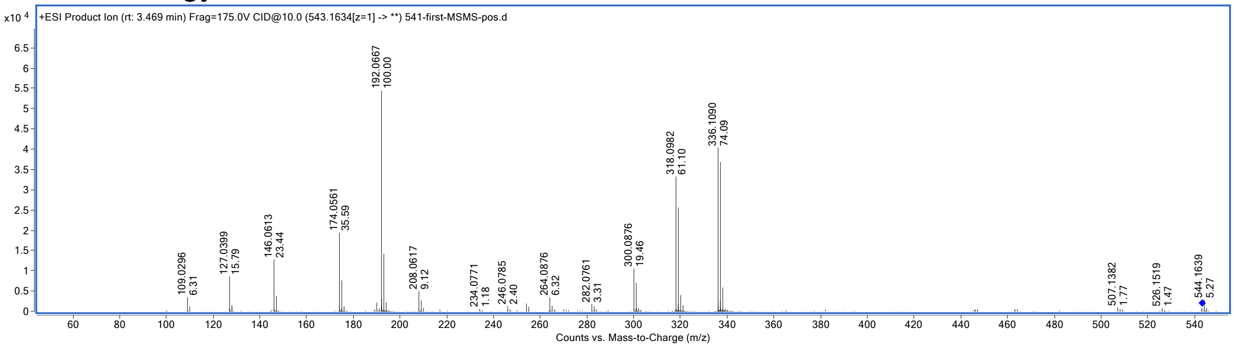

Collision Energy: 20eV

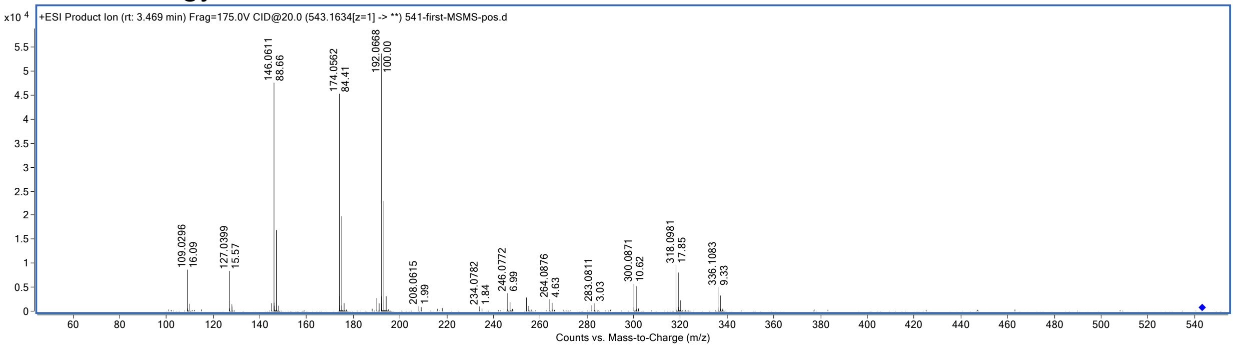

h) 2-(3-hydroxy-2-oxoindolin-3-l) acetic acid 3-O-6′- glucopyranosyl-2″-(2″oxoindolin-3″yl) acetate (Isomer 2)

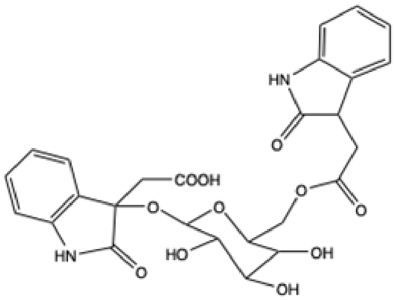

Collision Energy: 5eV

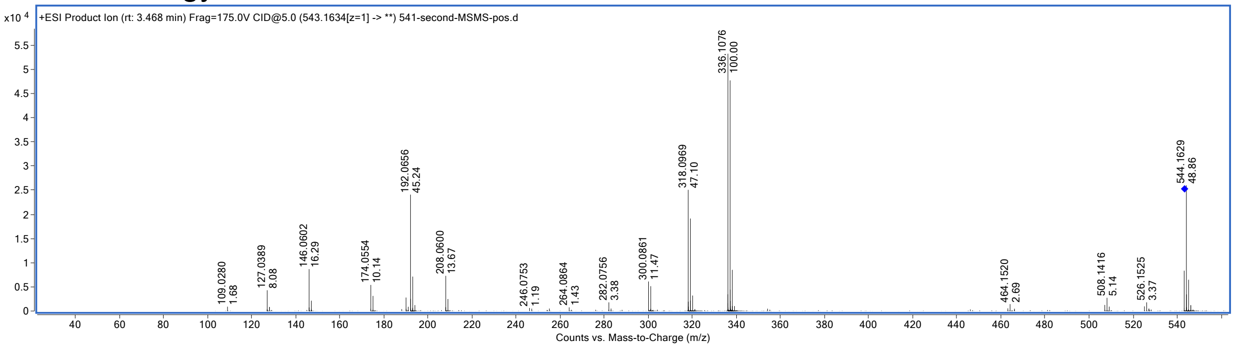

Collision Energy: 10eV

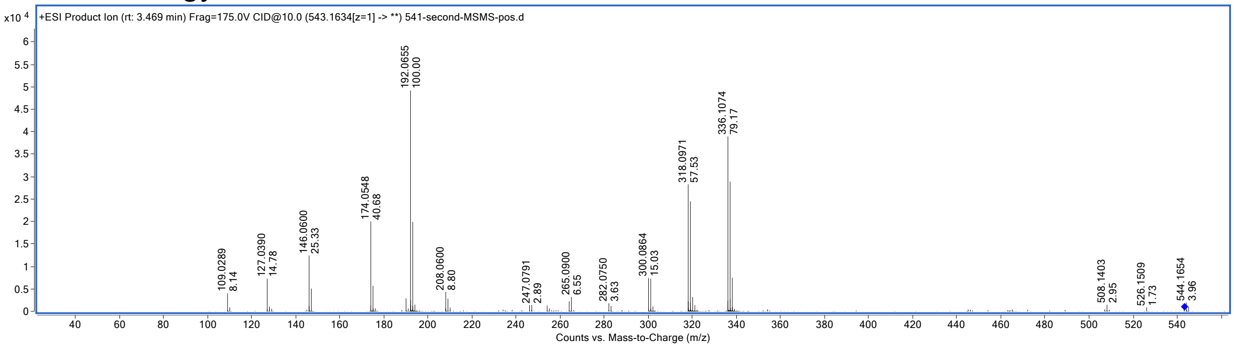

Collision Energy: 20eV

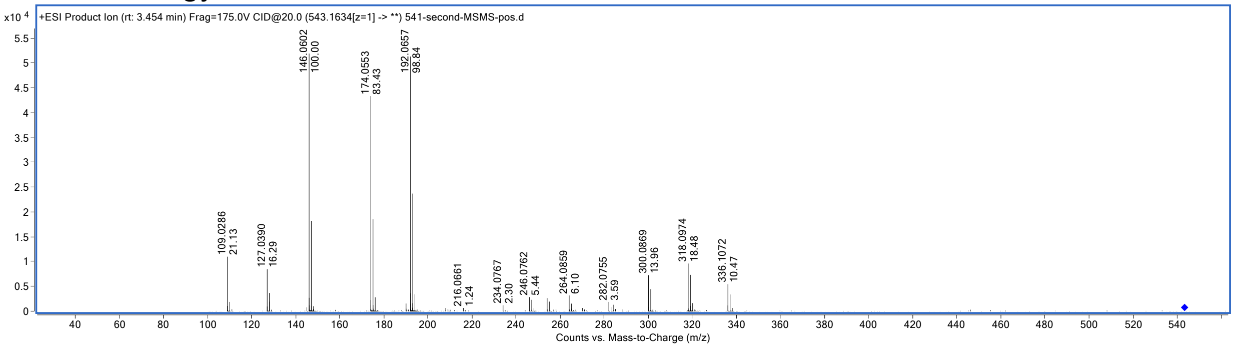

i) 1″-O-3′- *b* -glucofuranosyl-1’-O-1- *b* -glucofuranosyl-(2,6-dihydroxyphenyl)-ethan-4-one

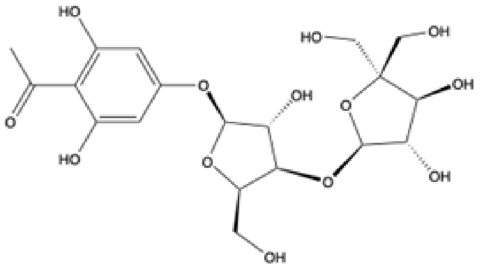

Collision Energy: 5eV

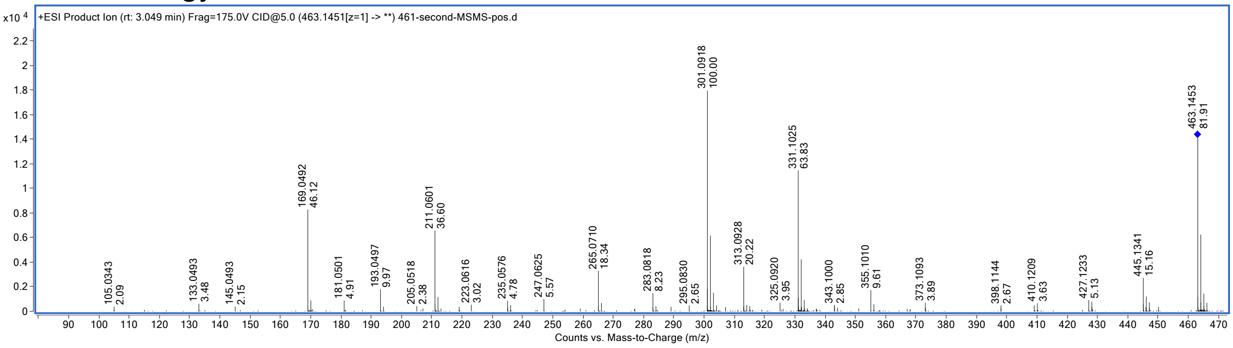

Collision Energy: 10eV

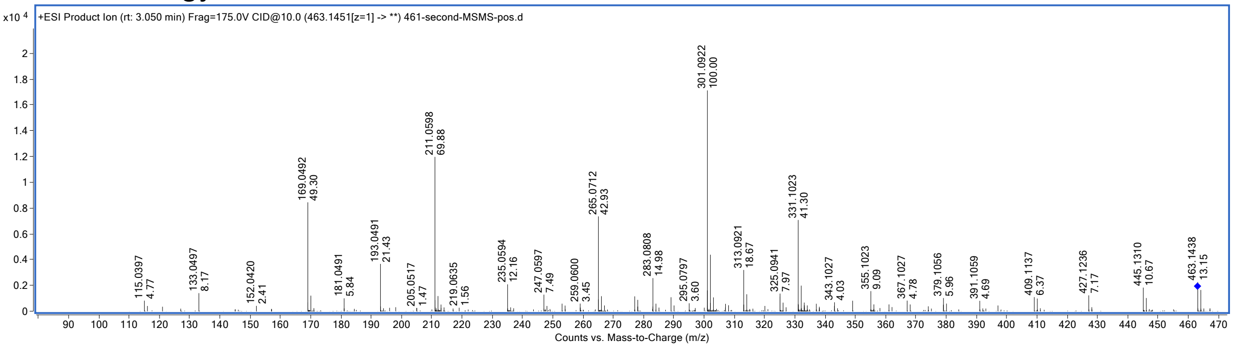

Collision Energy: 20eV

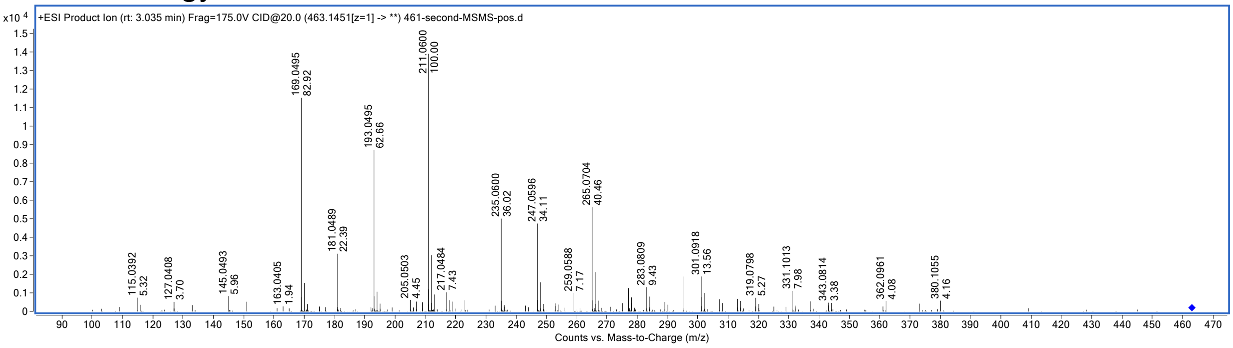

j) 5-Caffeoylquinic acid

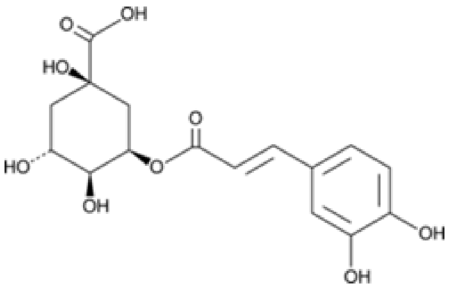

Collision Energy: 5eV

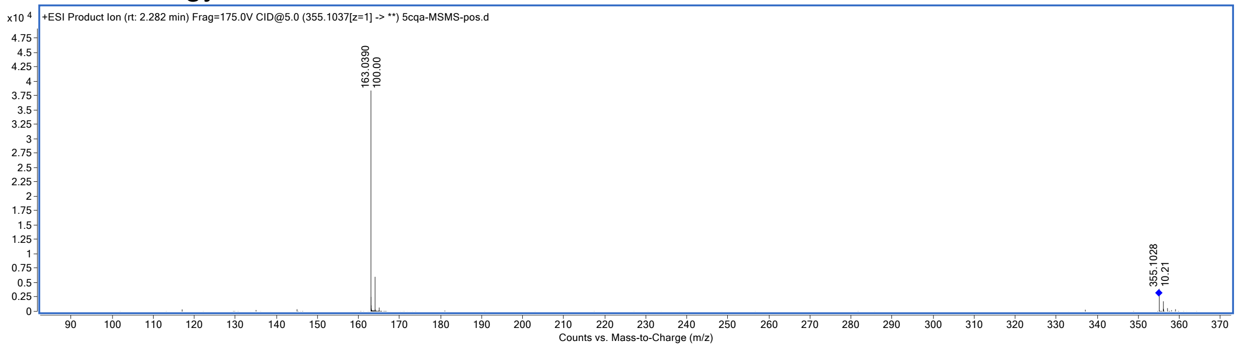

Collision Energy: 10eV

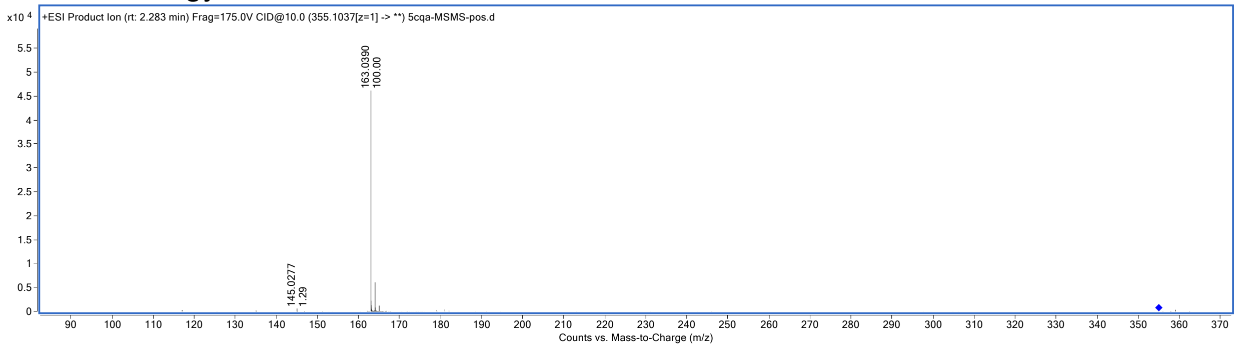

Collision Energy: 30eV

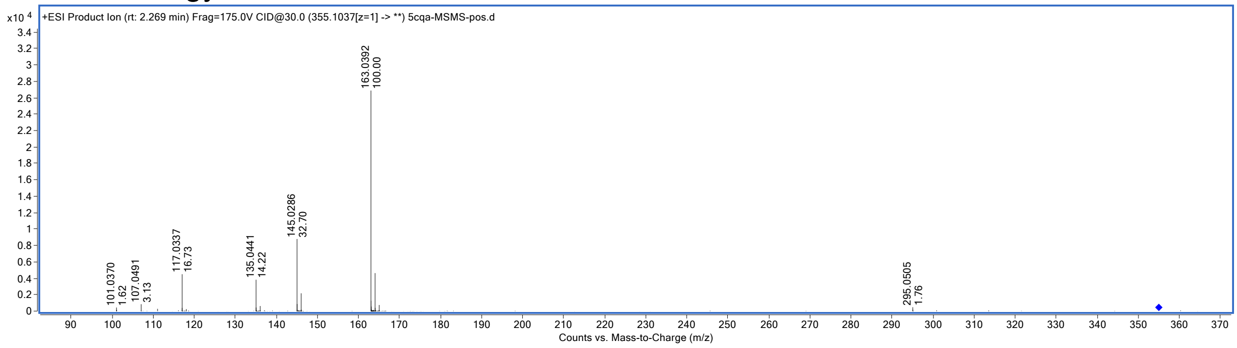

k) 4-Caffeoylquinic acid

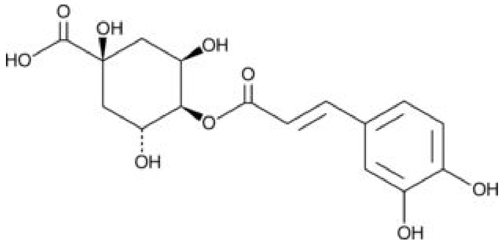

Collision Energy: 5eV

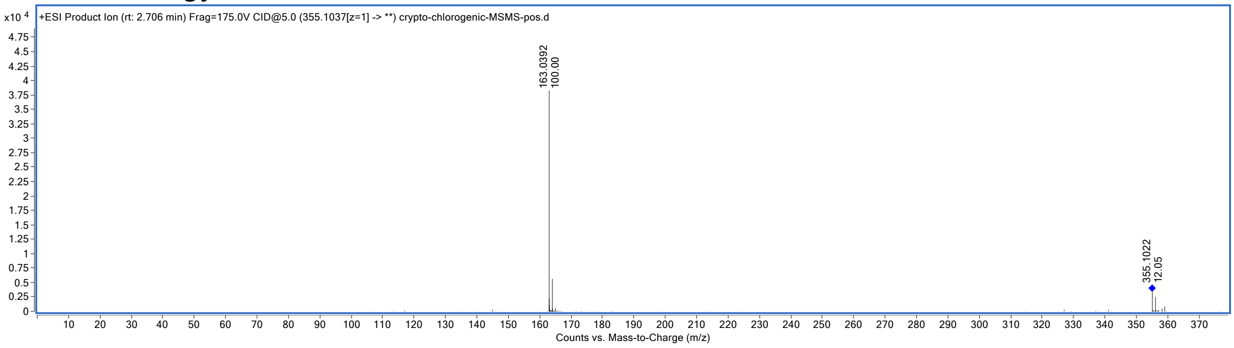

Collision Energy: 10eV

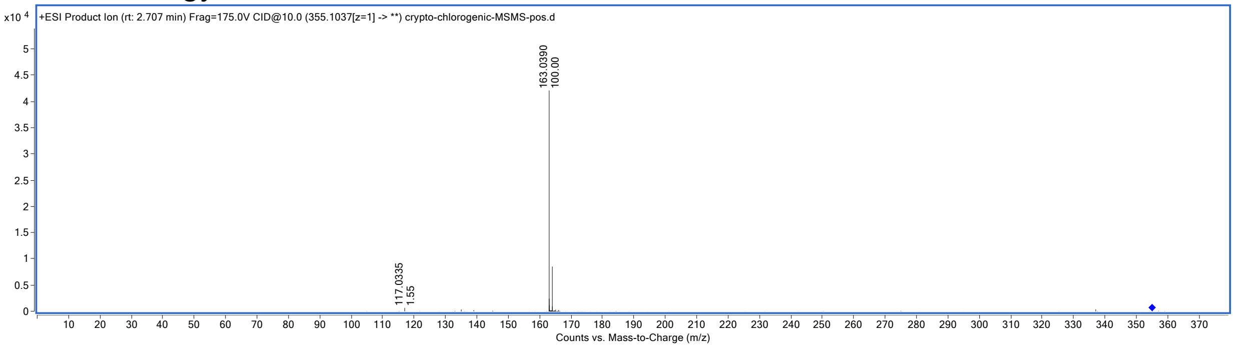

Collision Energy: 30eV

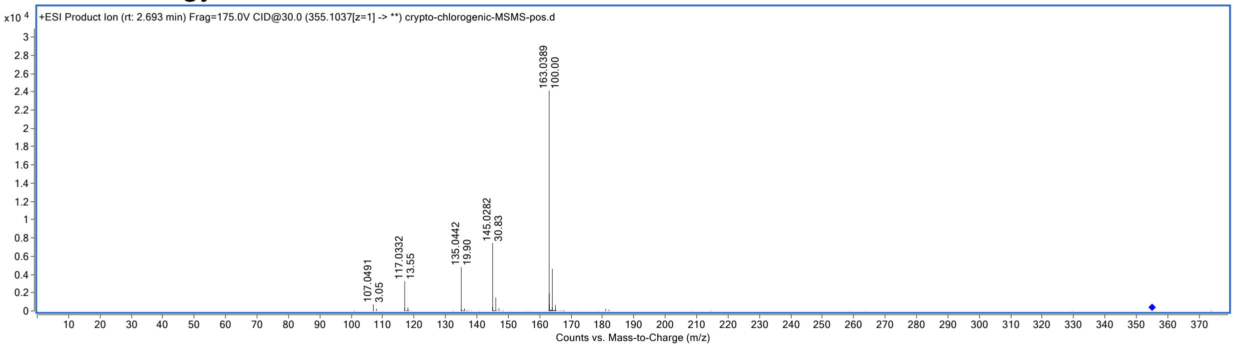

l) 1″-O-1′- *b* -glucofuranosyl-9-O-6′- *b* -glucopyranosyl-2″-(2″oxoindolin-3″yl) acetate (Isomer 1)

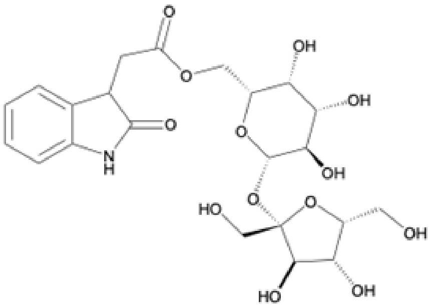

Collision Energy: 5eV

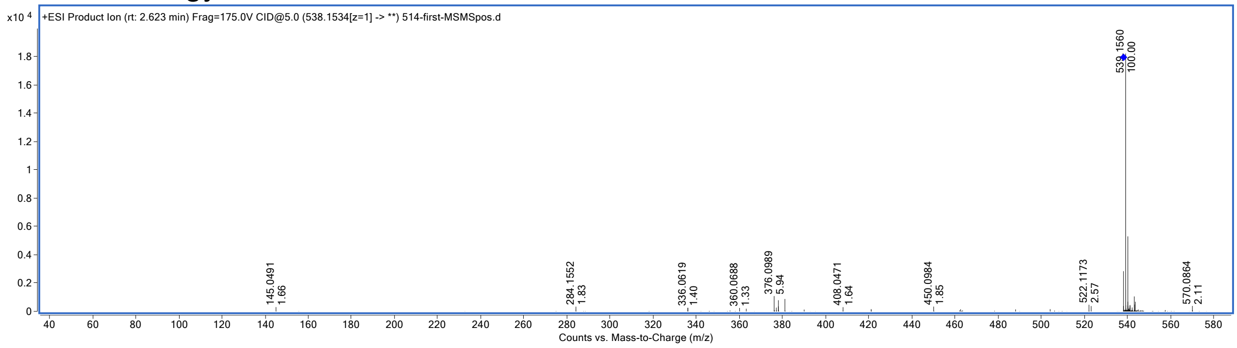

Collision Energy: 20eV

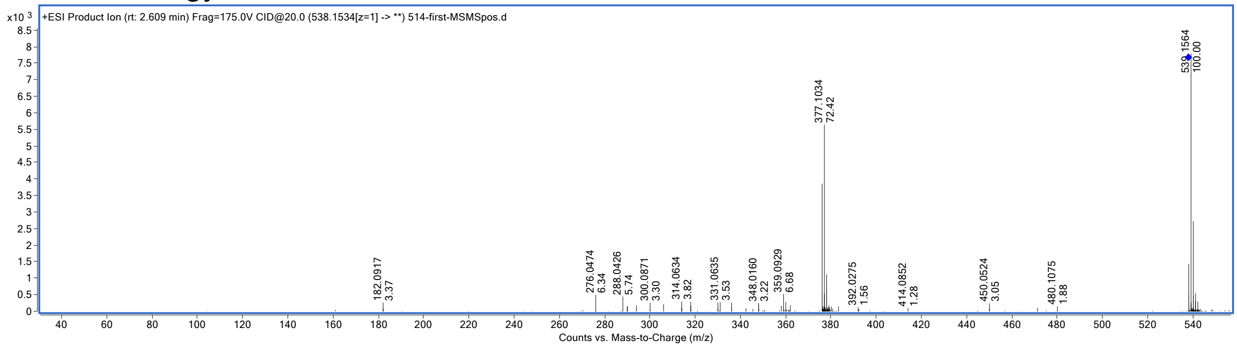

Collision Energy: 30eV

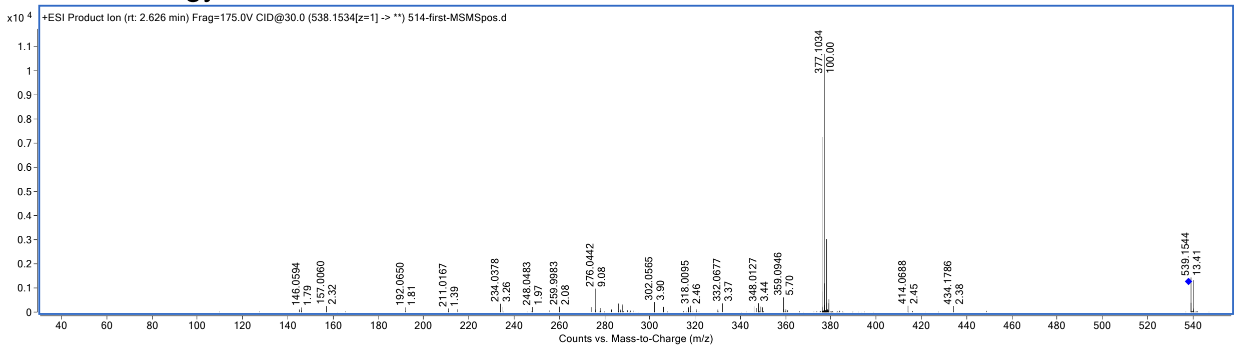

m) 1″-O-1′- *b* -glucofuranosyl-9-O-6′- *b* -glucopyranosyl-2″-(2″oxoindolin-3″yl) acetate (Isomer 2)

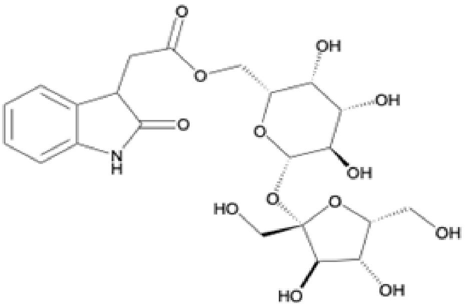

Collision Energy: 5eV

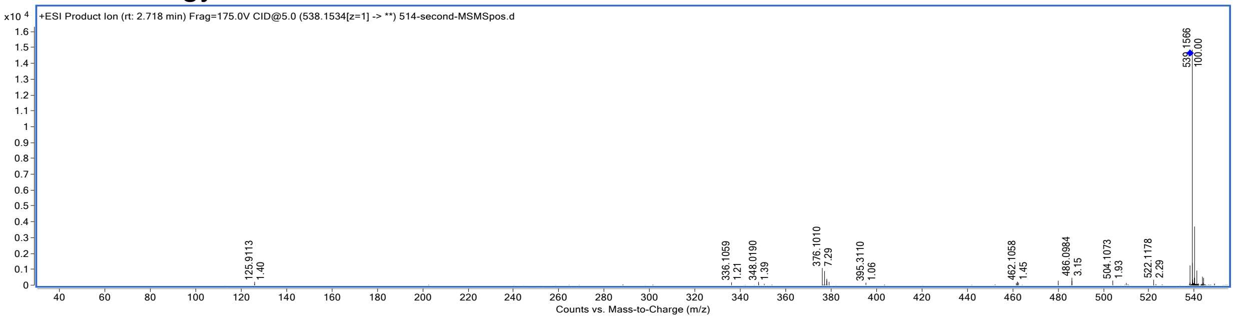

Collision Energy: 10eV

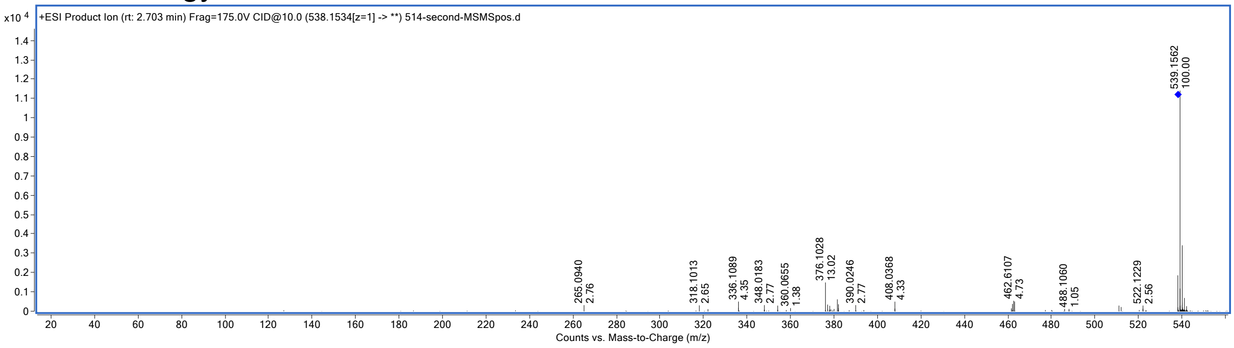

Collision Energy: 30eV

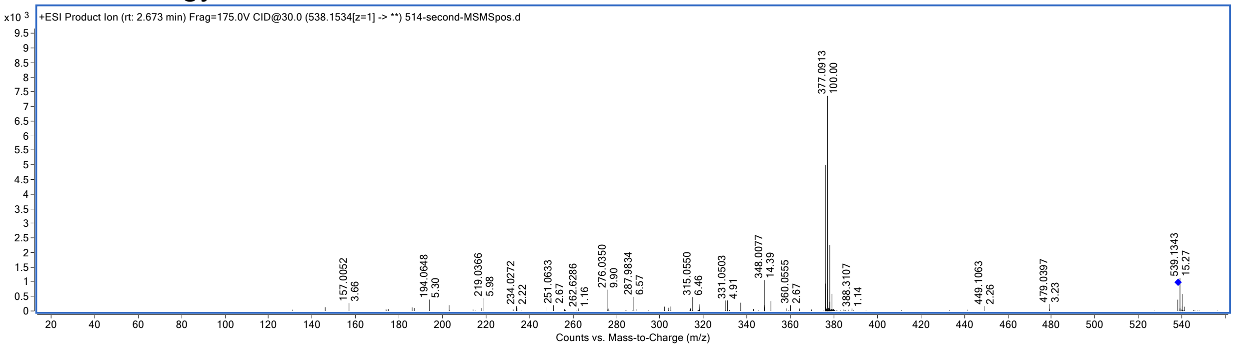

n) Caffeic acid

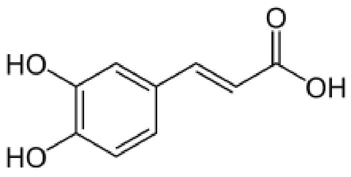

Collision Energy: 5eV

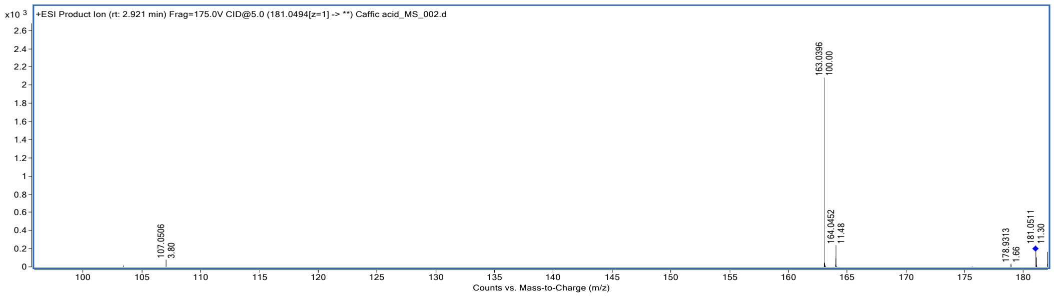

Collision Energy: 10eV

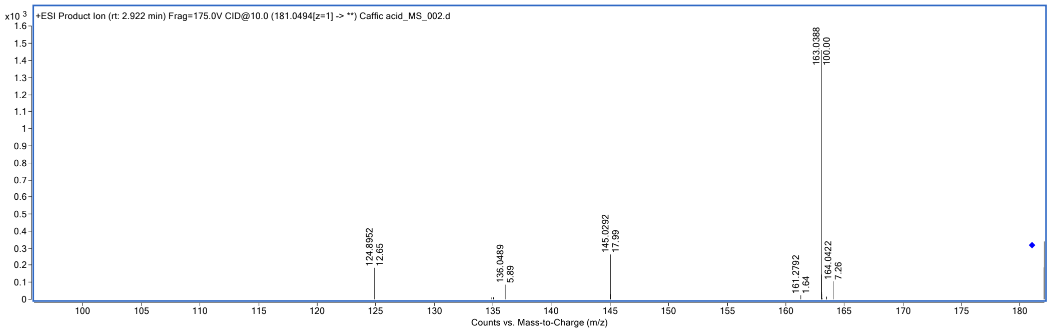

Collision Energy: 20eV

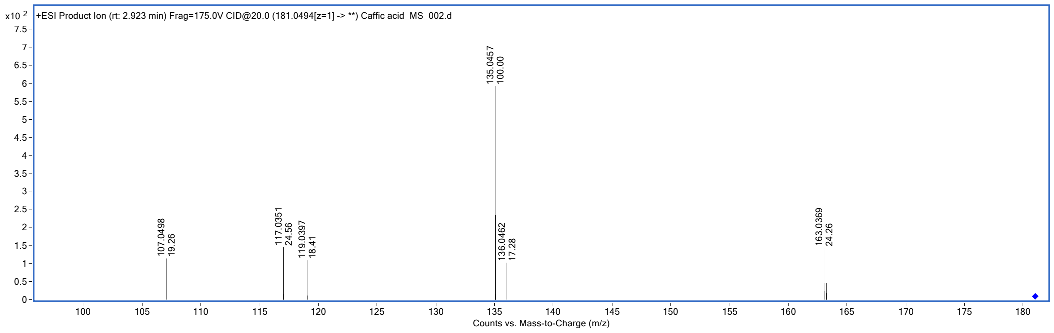

o) Genistein

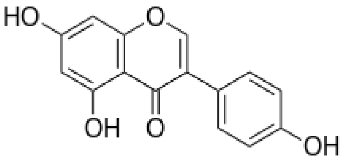

Collision Energy: 5eV

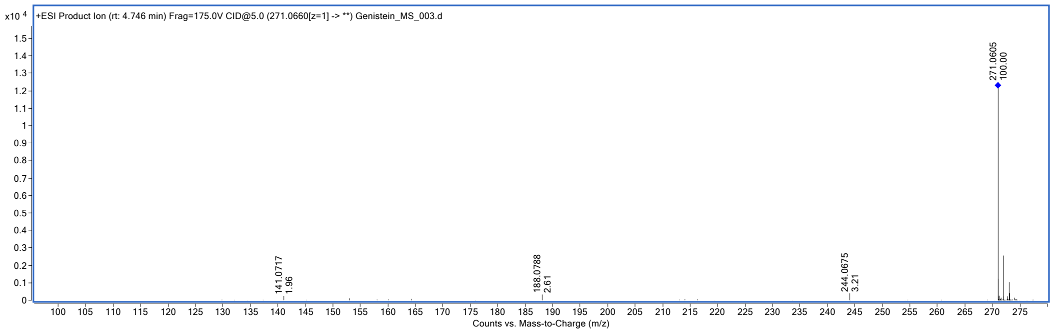

Collision Energy: 10eV

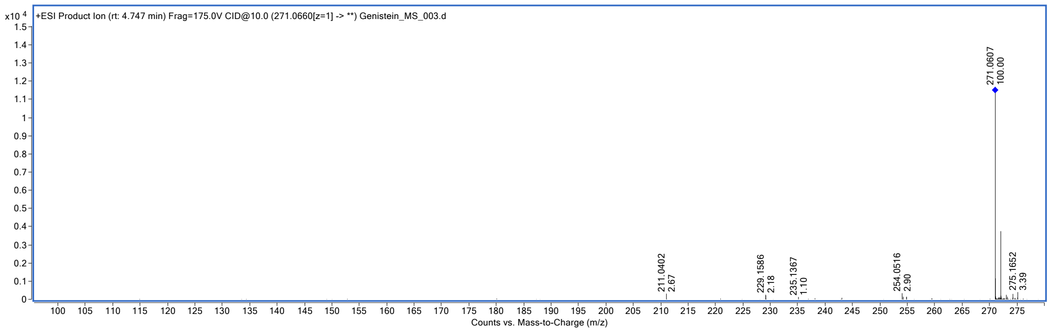

Collision Energy: 20eV

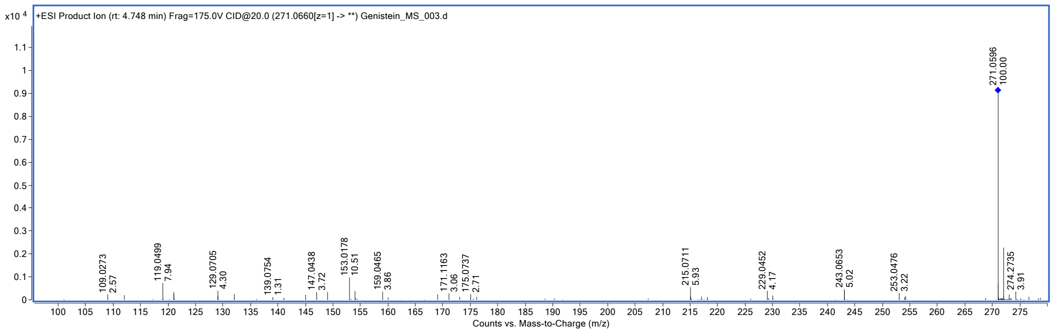

Collision Energy: 30eV

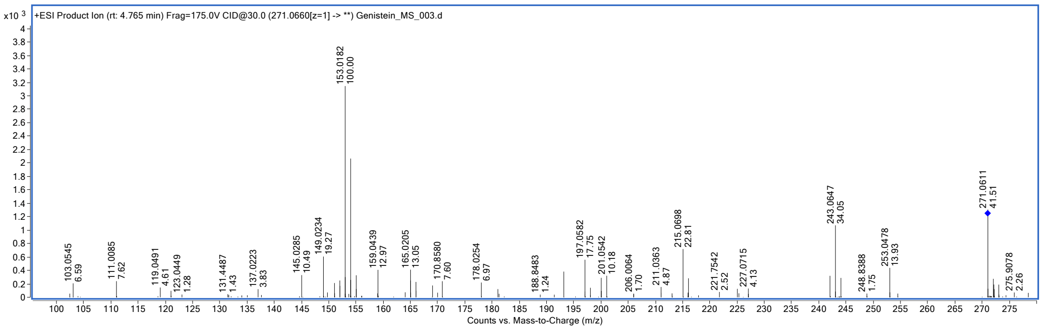

Collision Energy: 50eV

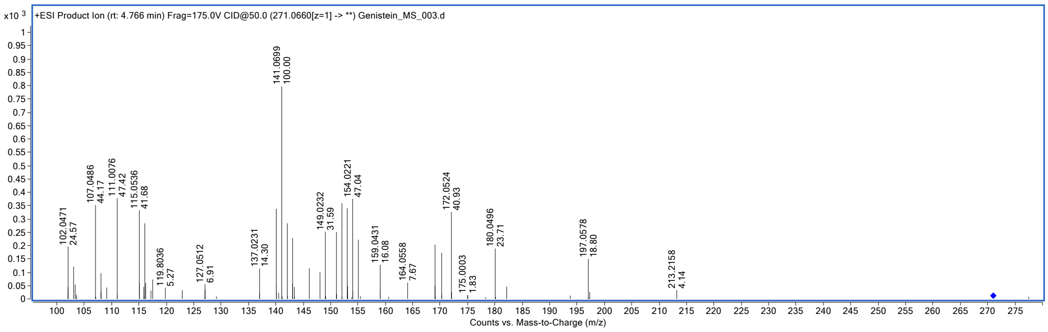

p) Daidzein

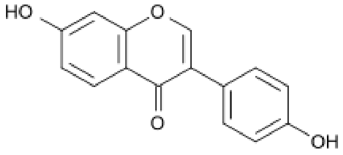

Collision Energy: 5eV

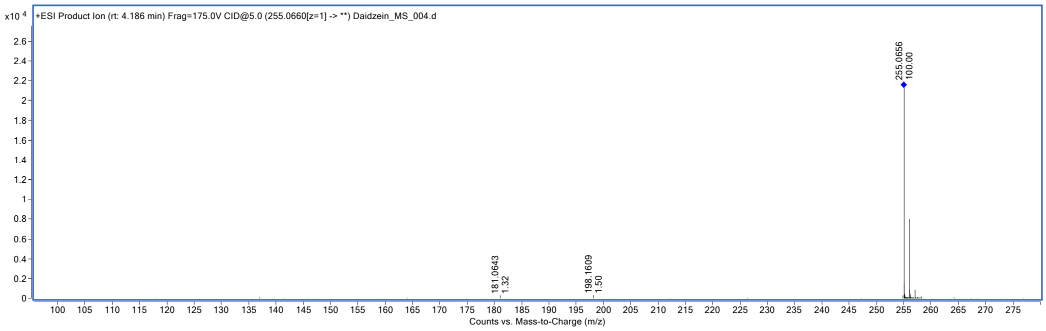

Collision Energy: 10eV

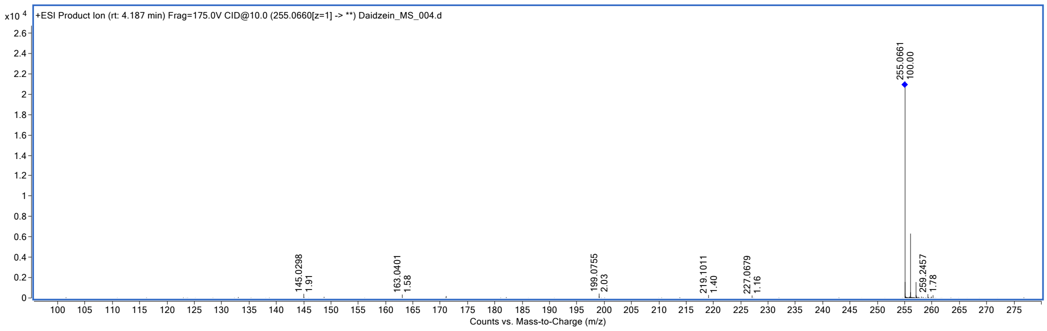

Collision Energy: 20eV

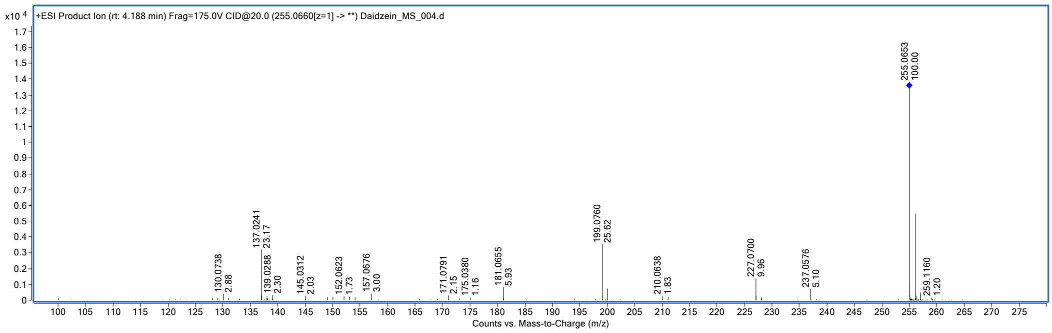

Collision Energy: 30eV

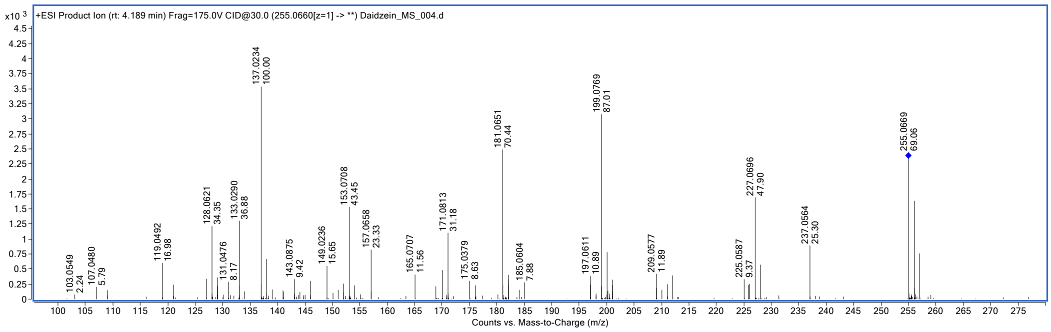

Collision Energy: 50eV

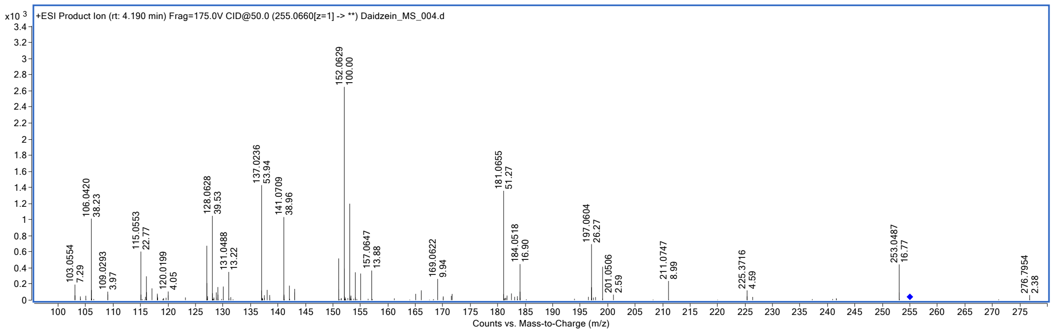

q) Soyasaponin 1

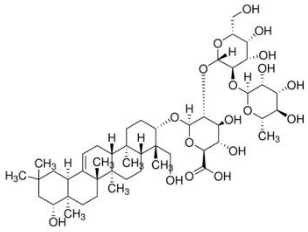

Collision Energy: 5eV

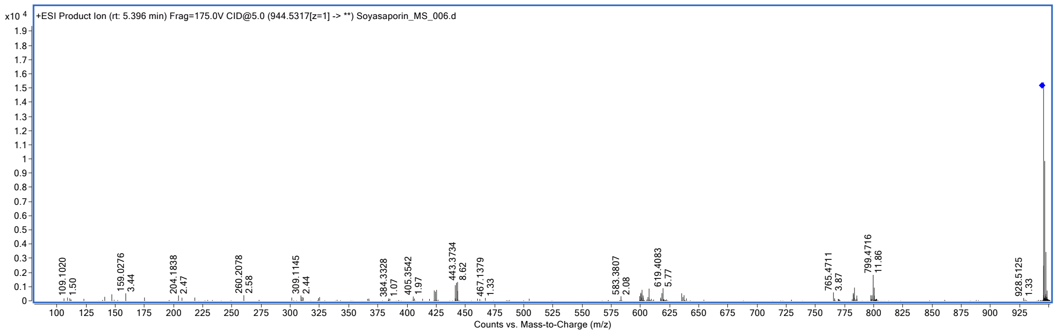

Collision Energy: 10eV

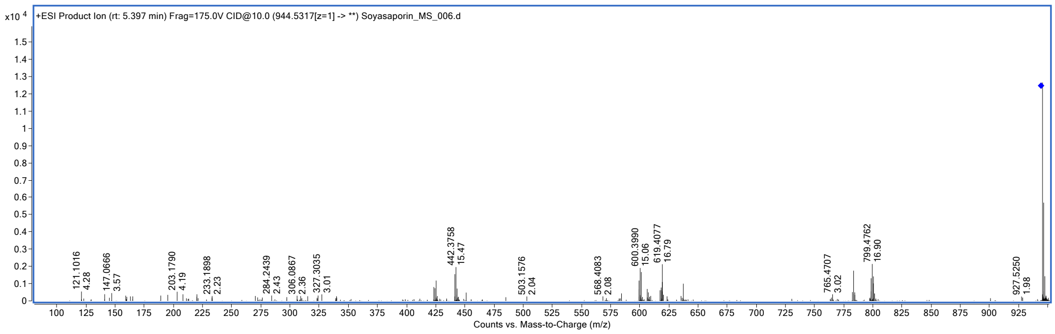

Collision Energy: 20eV

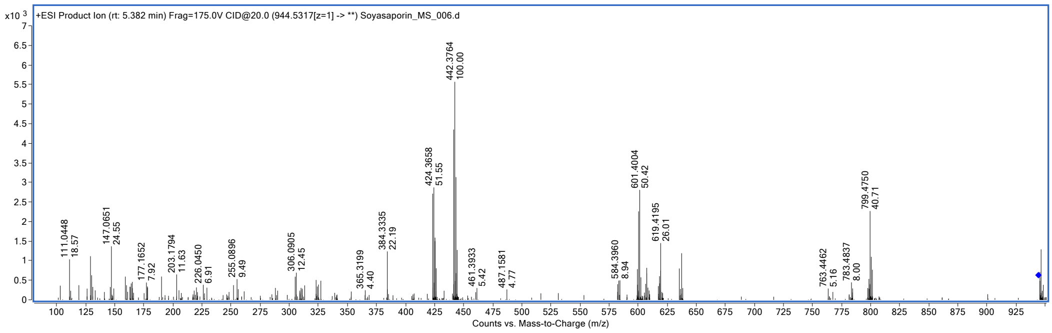

Collision Energy: 30eV

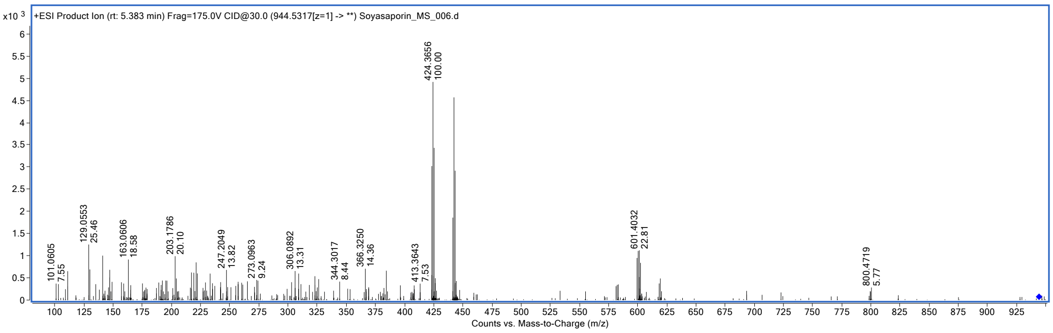

r) Pyroglutamic acid

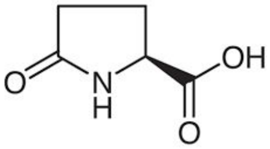

Collision Energy: 5eV

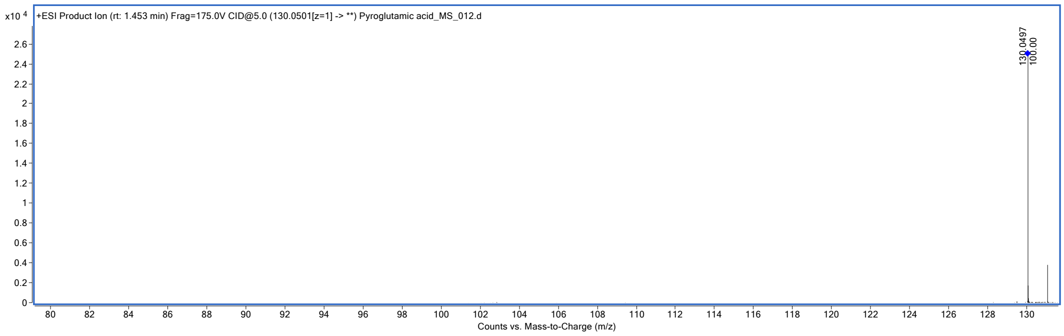

Collision Energy: 10eV

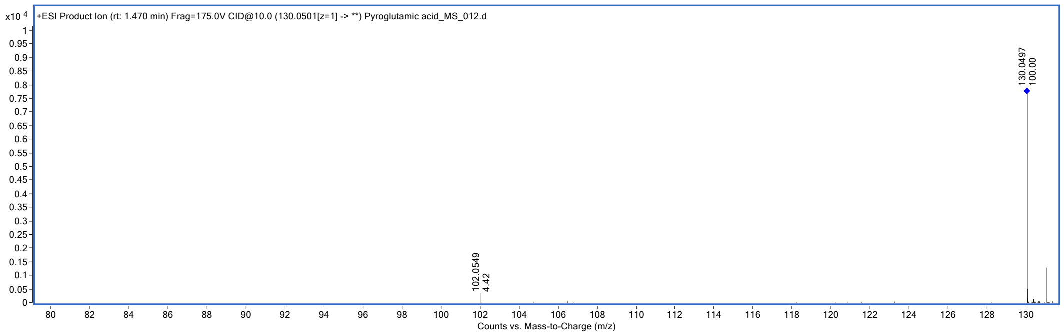

Collision Energy: 20eV

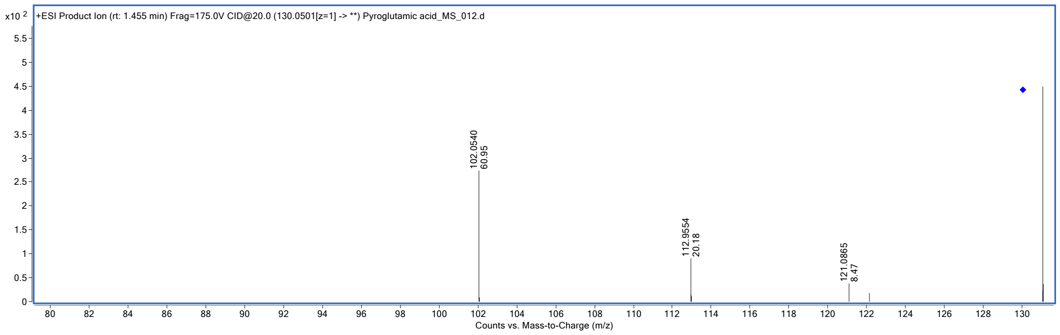

Collision Energy: 30eV

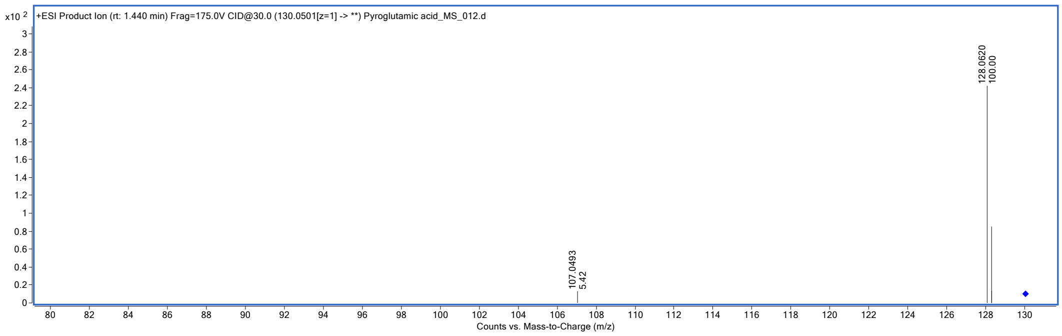

s) Rebaudioside A

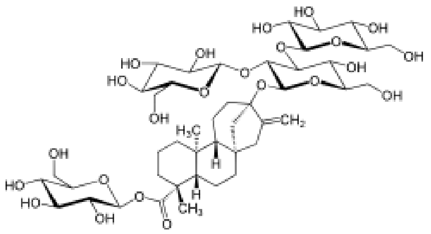

Collision Energy: 5eV

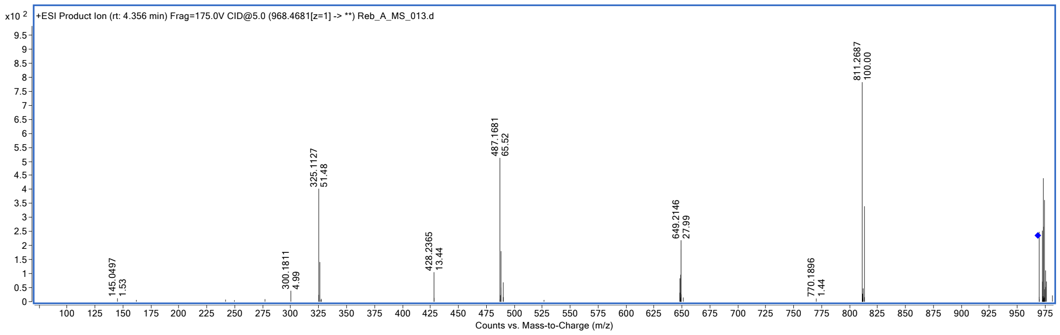

Collision Energy: 10eV

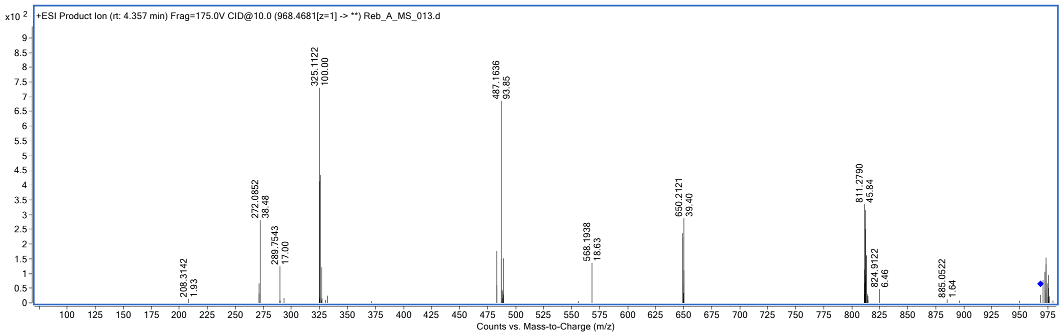

Collision Energy: 20eV

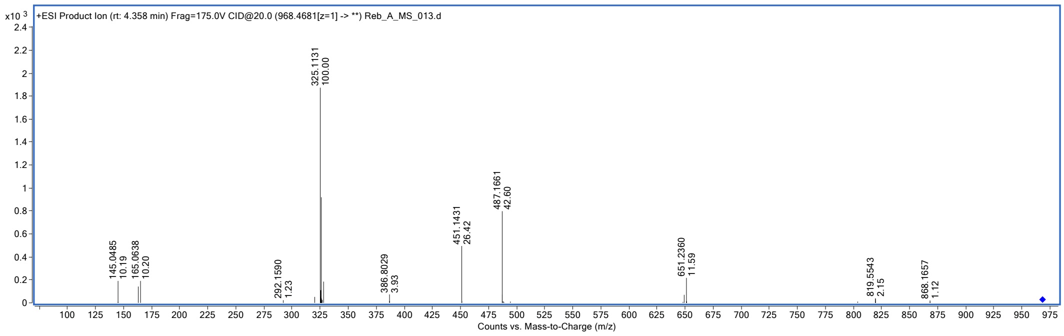

t) Rebaudioside A degradation compound 1

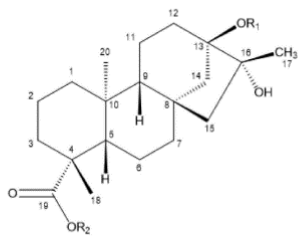

R1: Glc 1–2(Glcβ1-3) Glcβ1, R2: Glcβ1

Collision Energy: 5eV

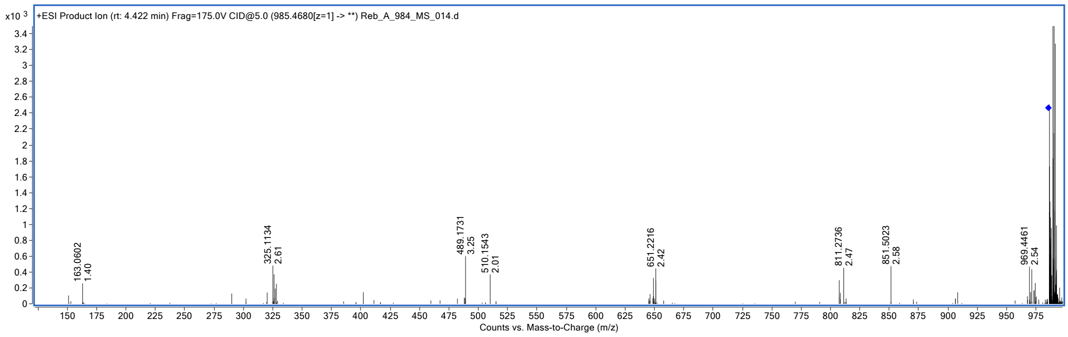

Collision Energy: 10eV

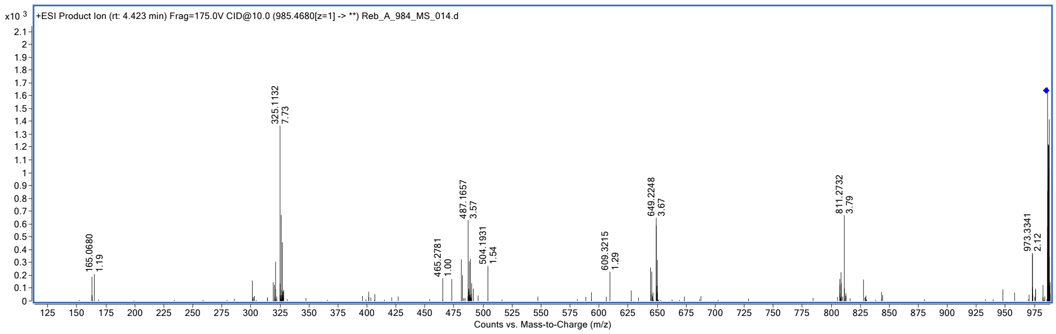

Collision Energy: 20eV

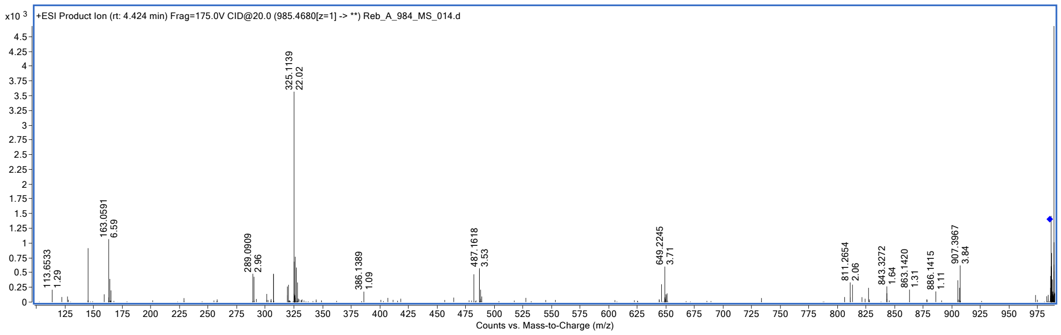

Collision Energy: 30eV

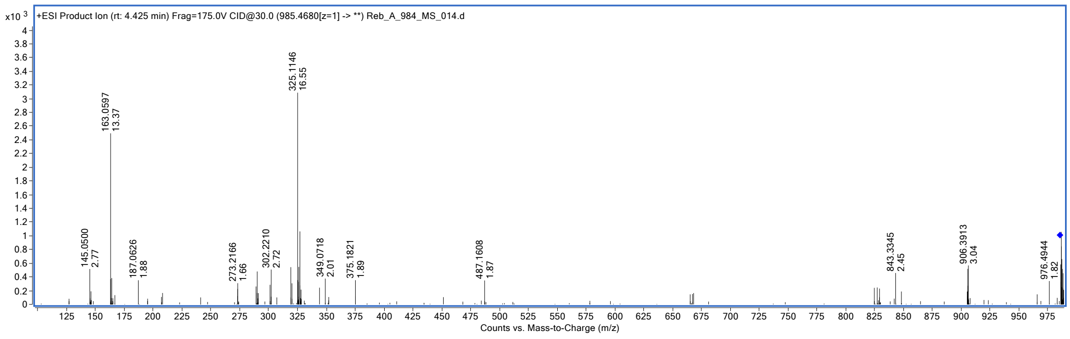

Collision Energy: 50e

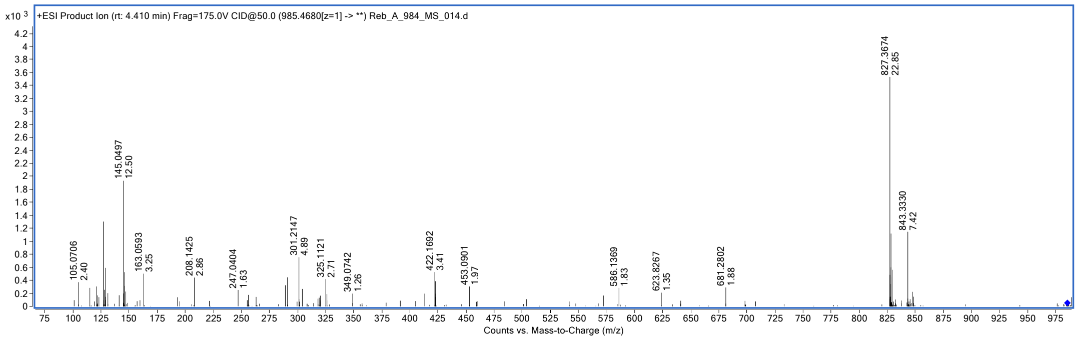

u) Rebaudioside A degradation compound 2

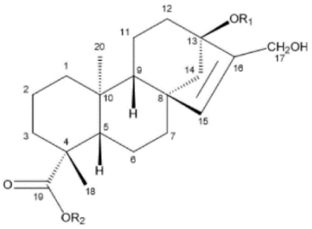

R1: Glc 1–2(Glcβ1-3) Glcβ1, R2: Glcβ1

Collision Energy: 5eV

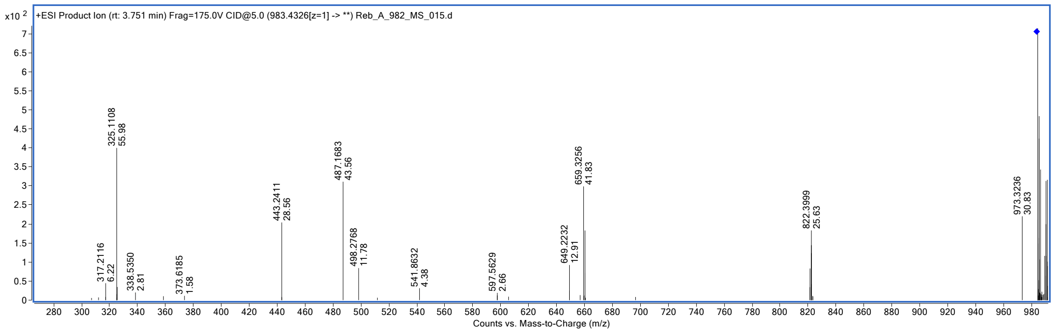

Collision Energy: 10eV

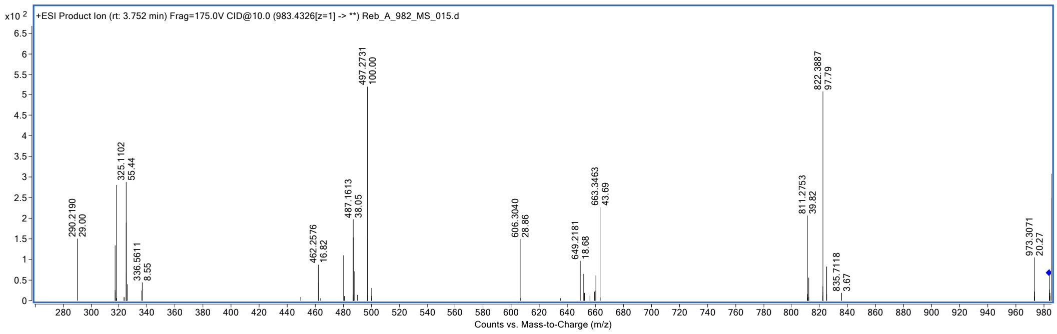

Collision Energy: 20eV

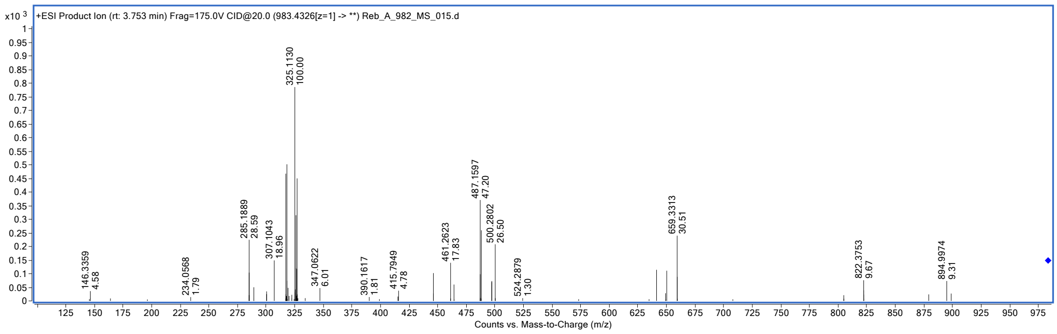

Collision Energy: 30eV

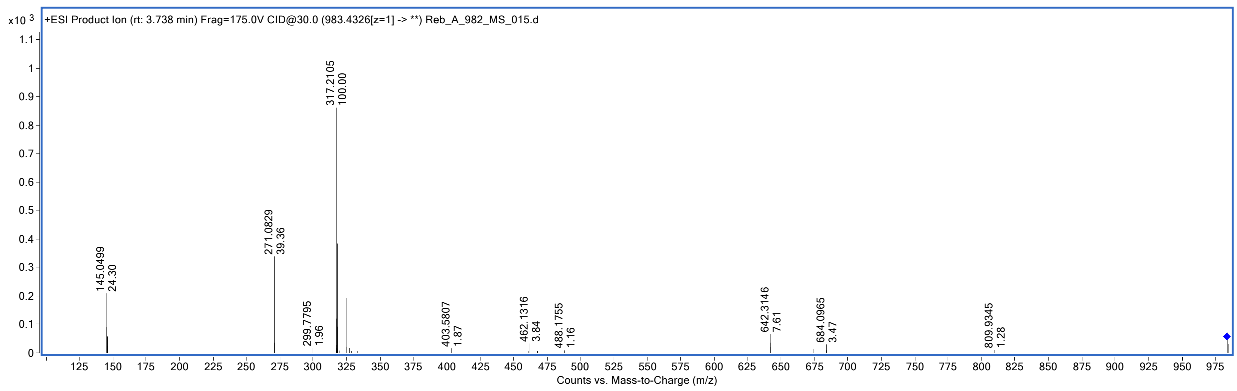

Collision Energy: 50eV

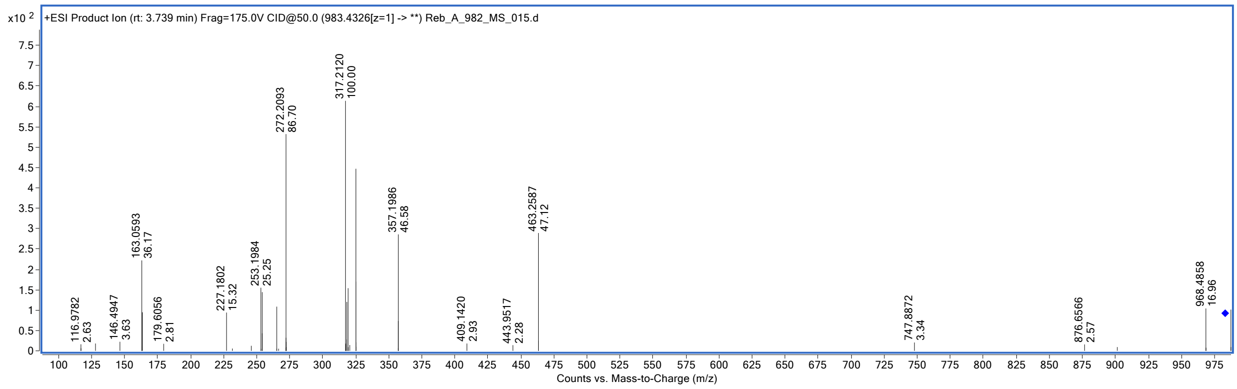

Collision Energy: 70eV

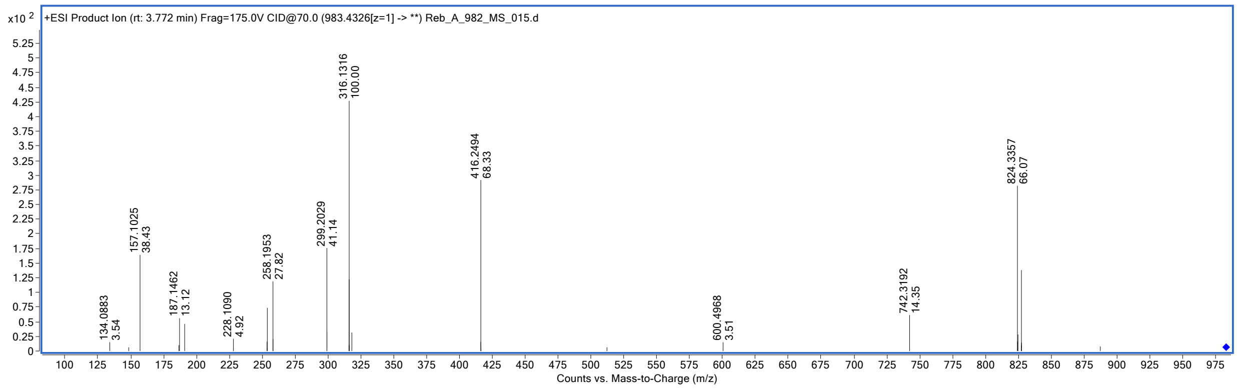

v) Rebaudioside A degradation compound 3

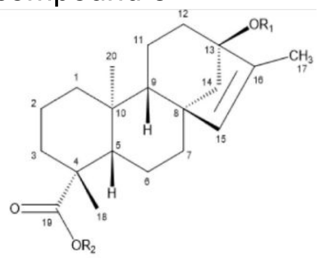

R1: Glc 1–2(Glcβ1-3) Glcβ1, R2: Glcβ1

Collision Energy: 5eV

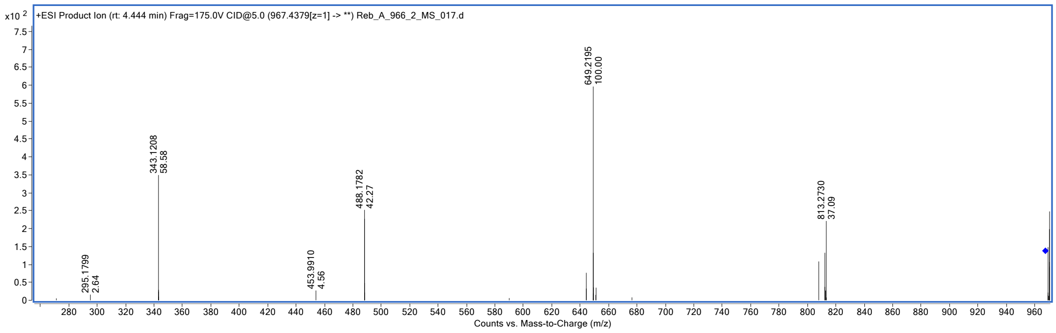

Collision Energy: 10eV

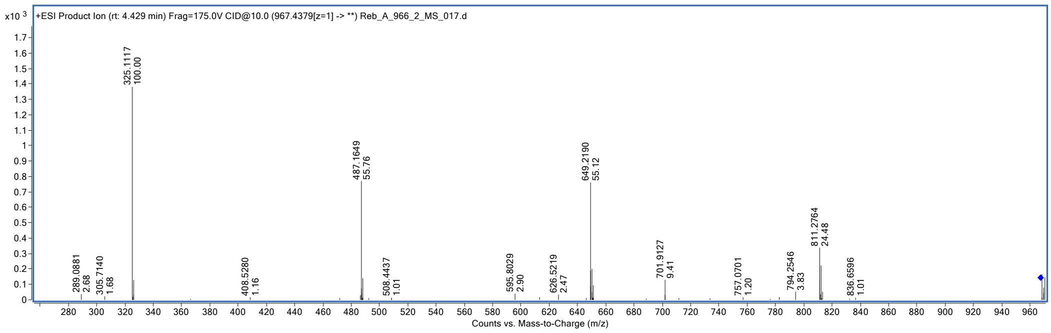

Collision Energy: 20eV

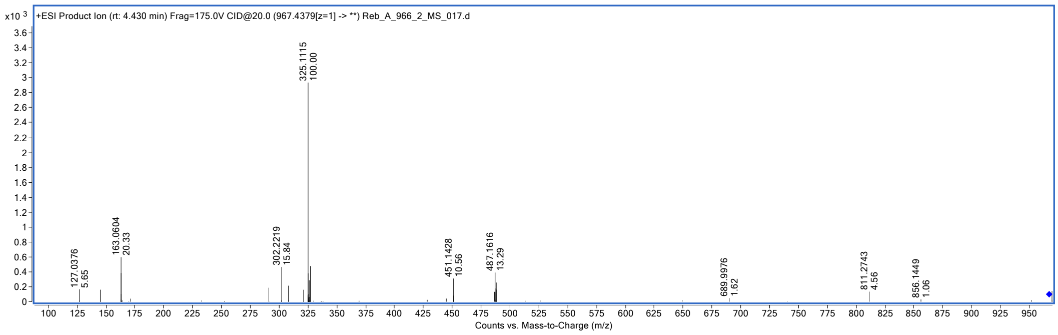

Collision Energy: 30eV

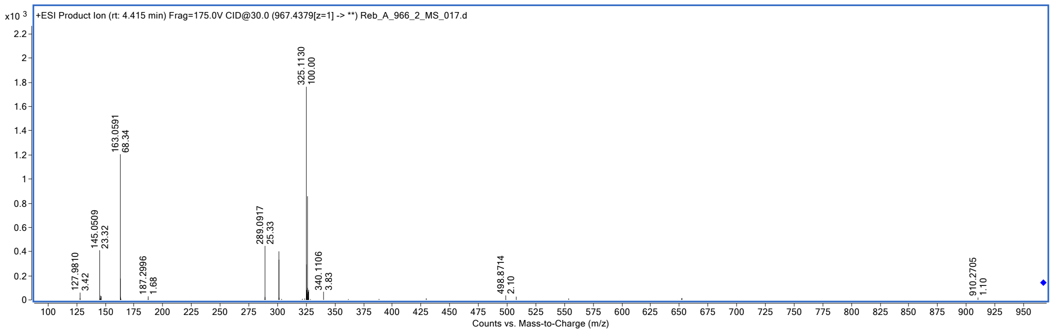

Collision Energy: 50eV

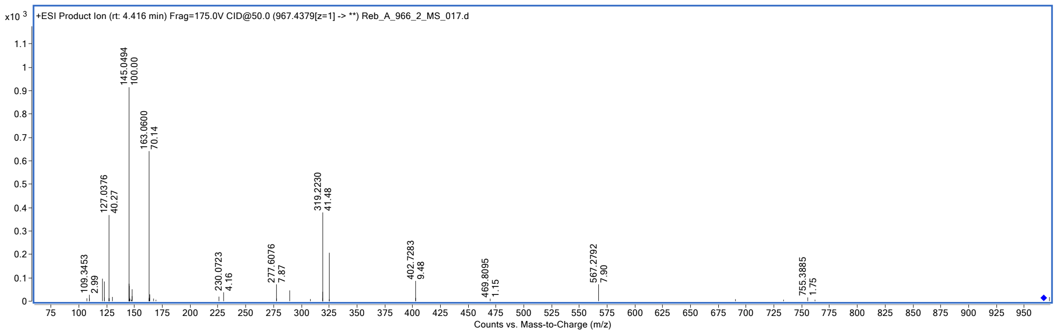

w) Inosine monophosphate

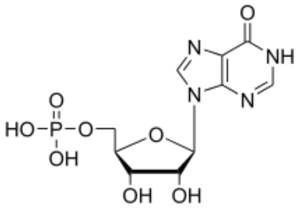

Collision Energy: 5eV

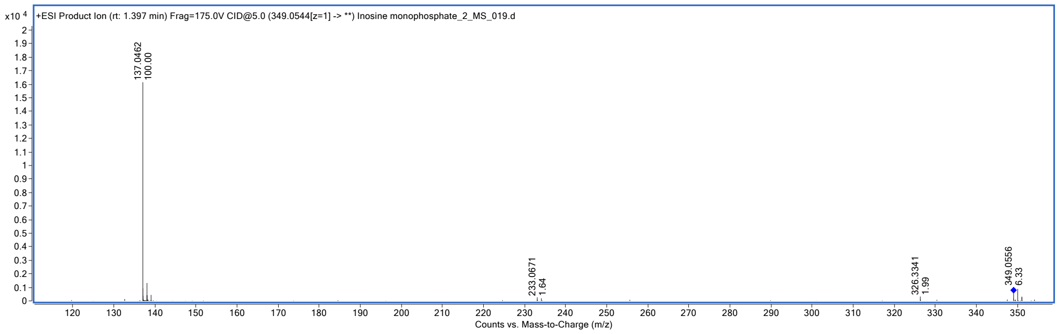

Collision Energy: 10eV

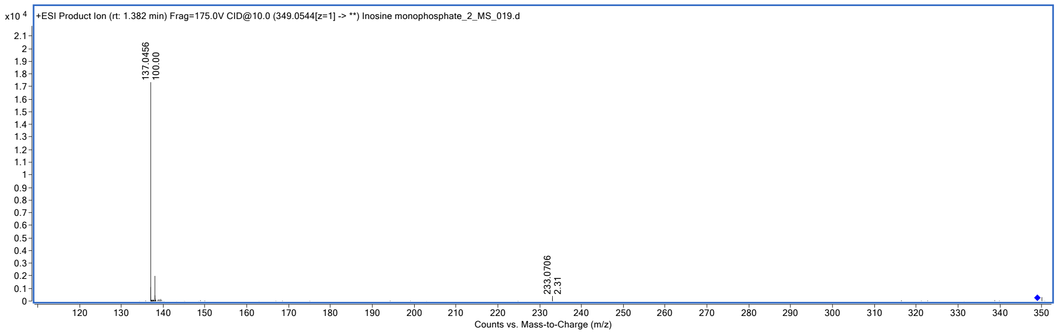

x) Adenosine monophosphate

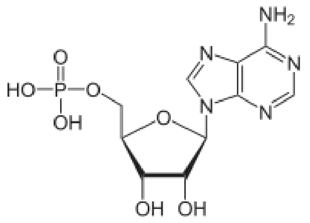

Collision Energy: 5eV

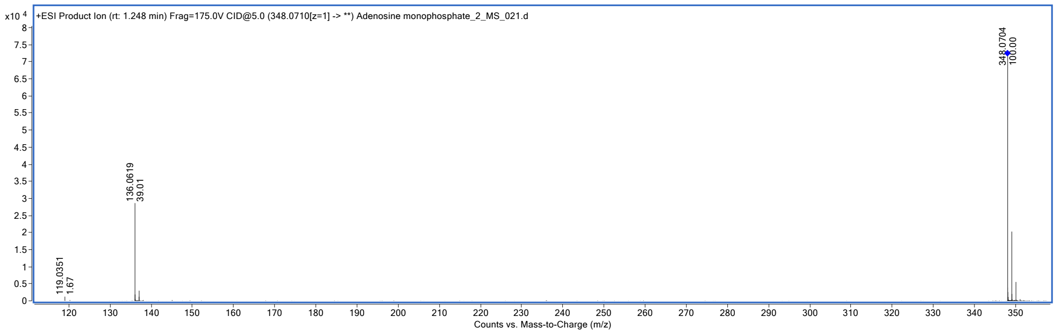

Collision Energy: 10eV

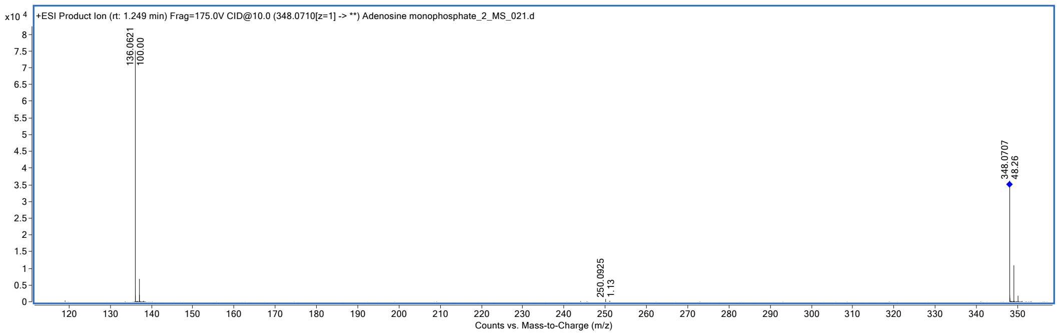

Collision Energy: 20eV

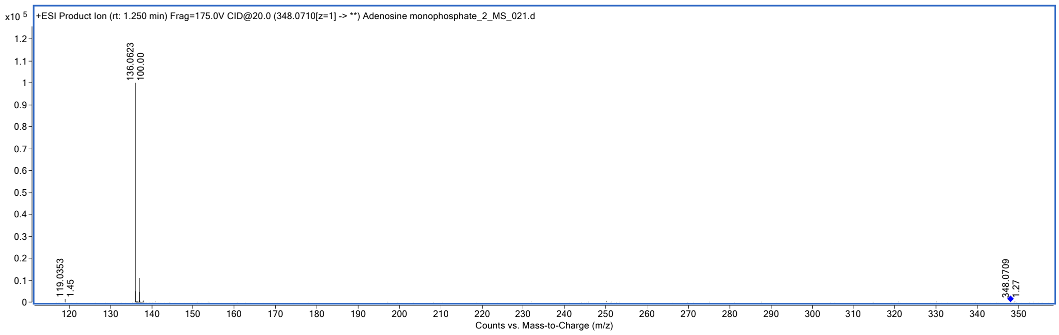

Collision Energy: 30eV

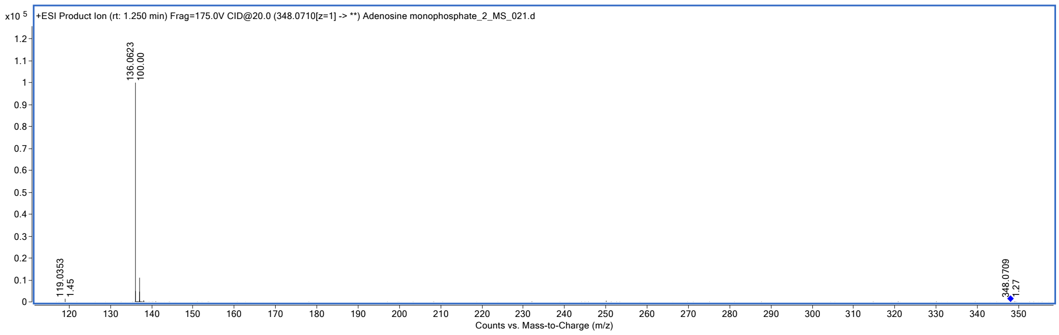

y) Uridine monophosphate

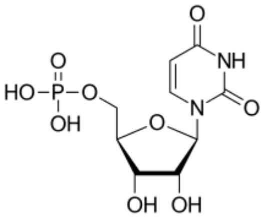

Collision Energy: 5eV

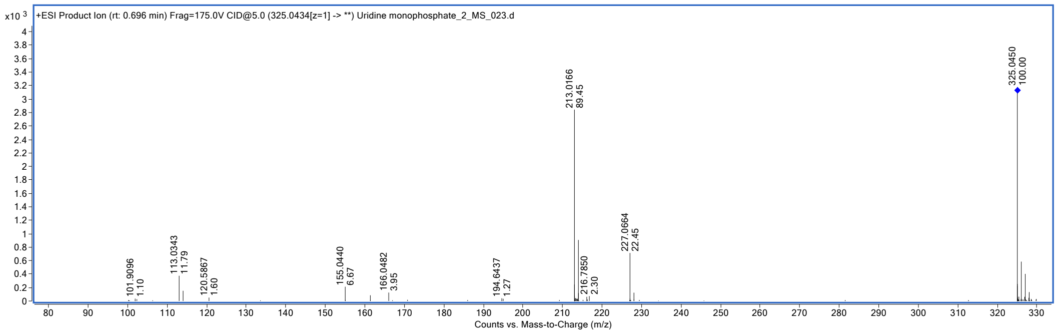

Collision Energy: 10eV

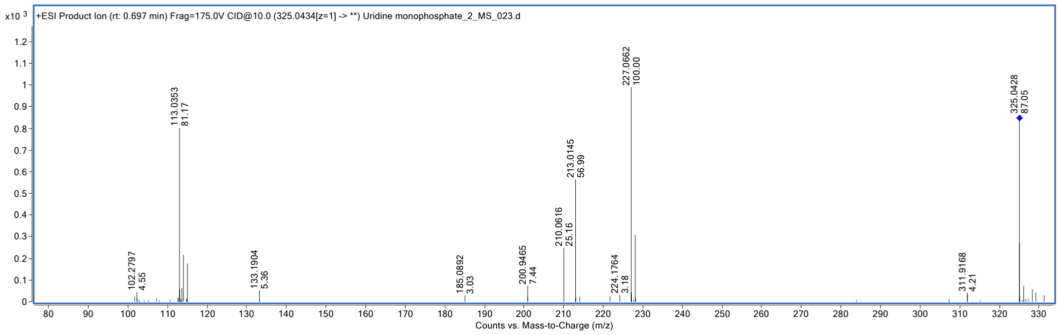

Collision Energy: 20eV

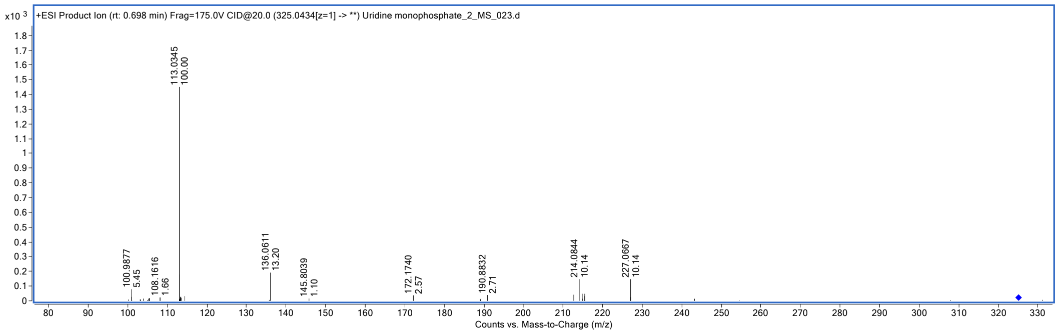

z) Guanosine monophosphate

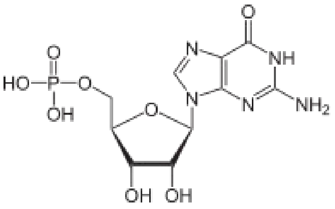

Collision Energy: 5eV

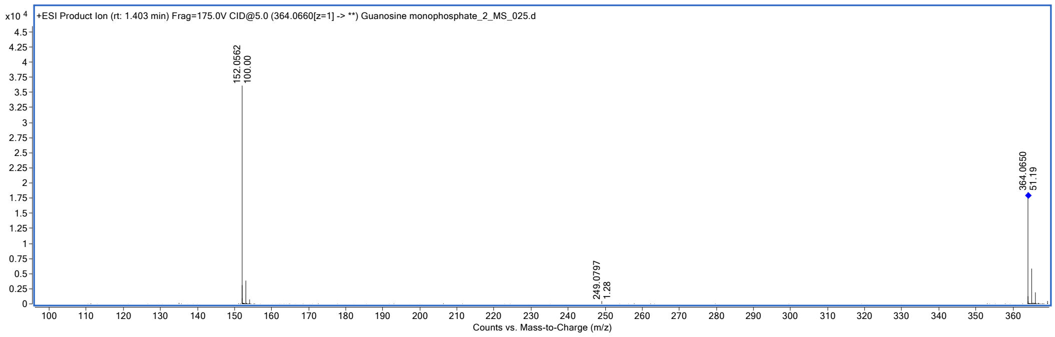

Collision Energy: 10eV

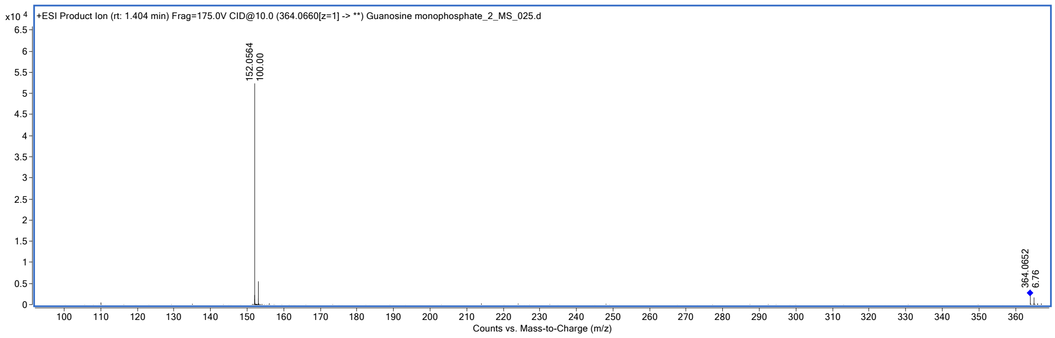

Collision Energy: 20eV

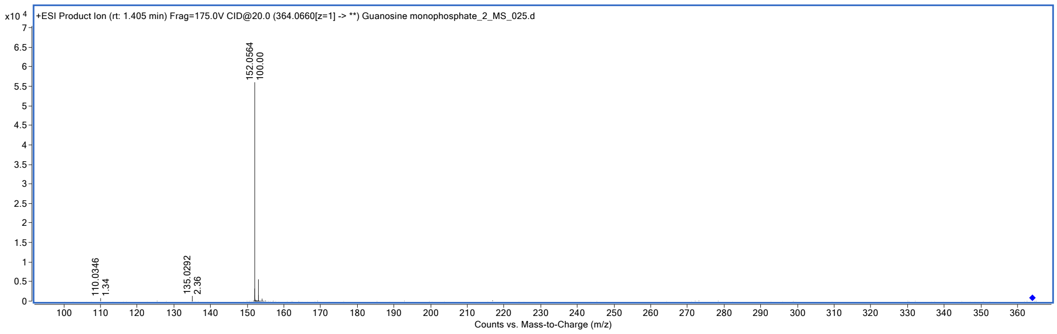

aa) Monosodium glutamate

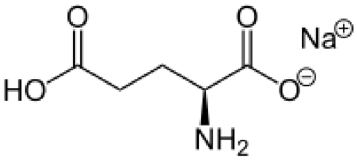

Collision Energy: 5eV

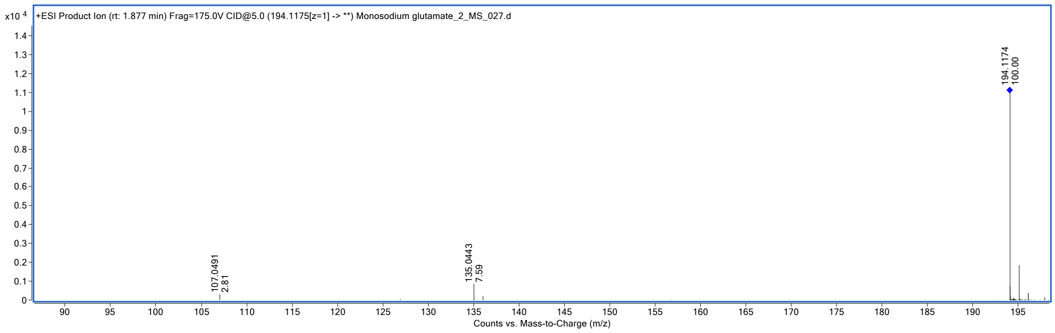

Collision Energy: 10eV

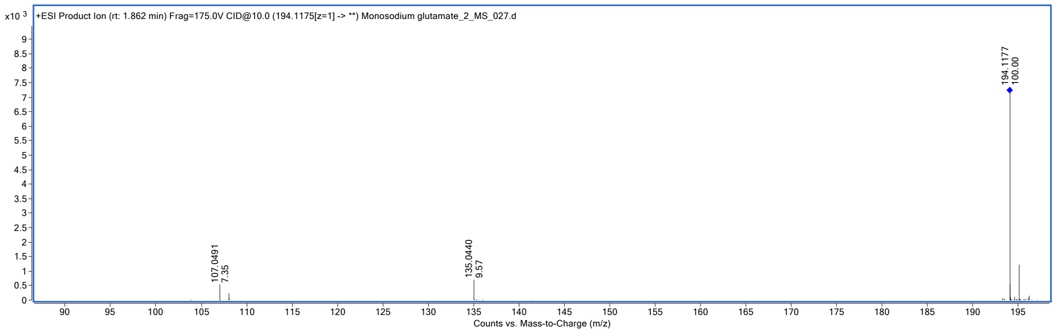

Collision Energy: 20eV

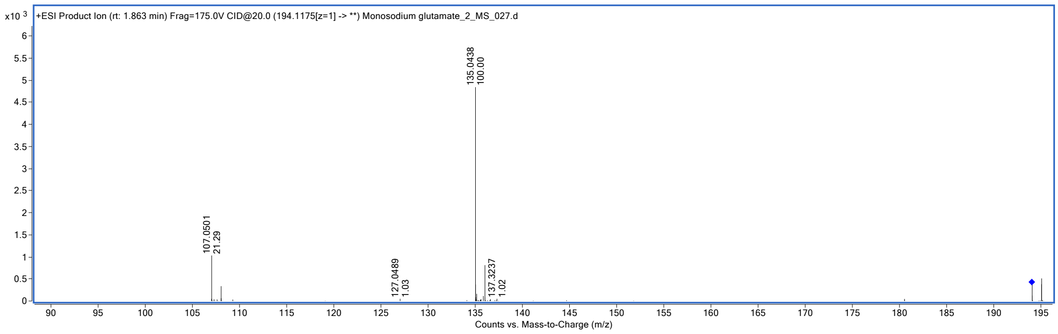

Collision Energy: 30eV

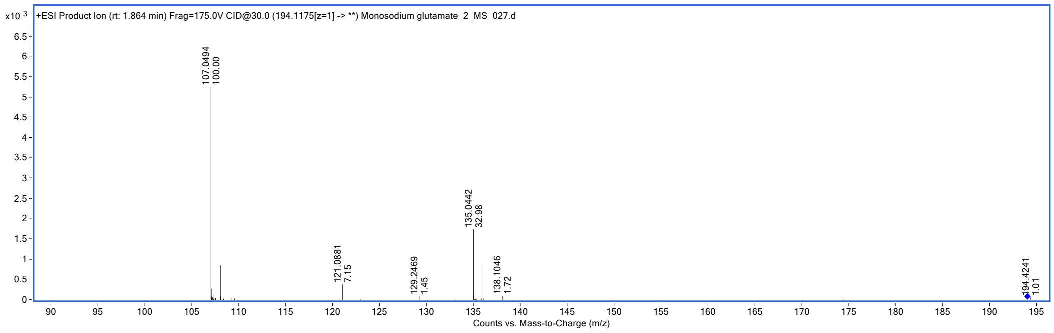

bb) 3-O-caffeoyl-4-O-3-methylbutanoylquinic acid

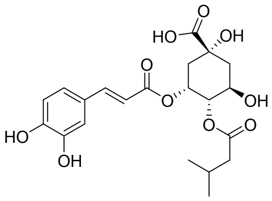

Collision Energy: 5eV

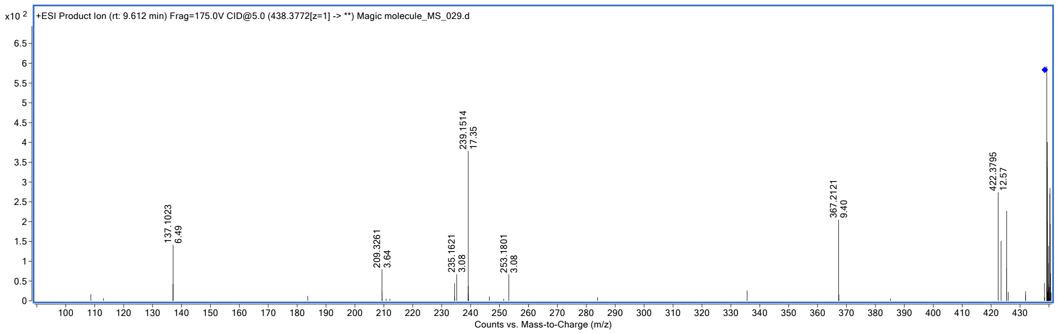

Collision Energy: 10eV

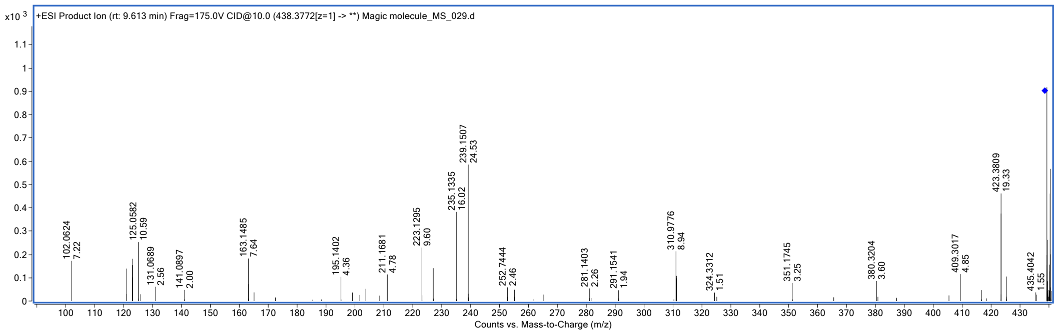

Collision Energy: 20eV

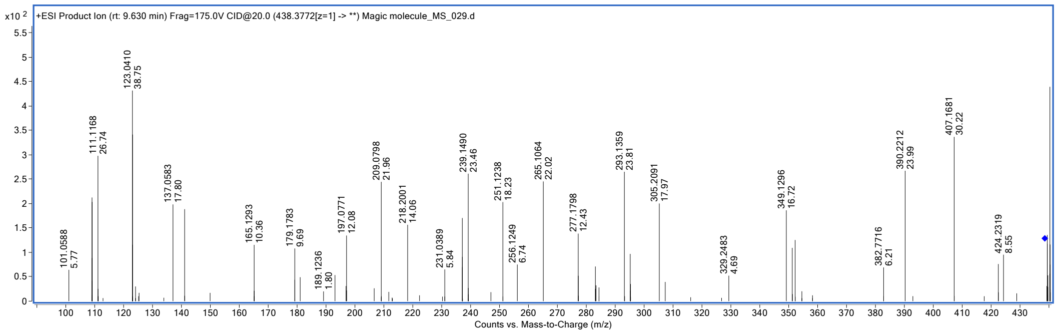

Collision Energy: 30eV

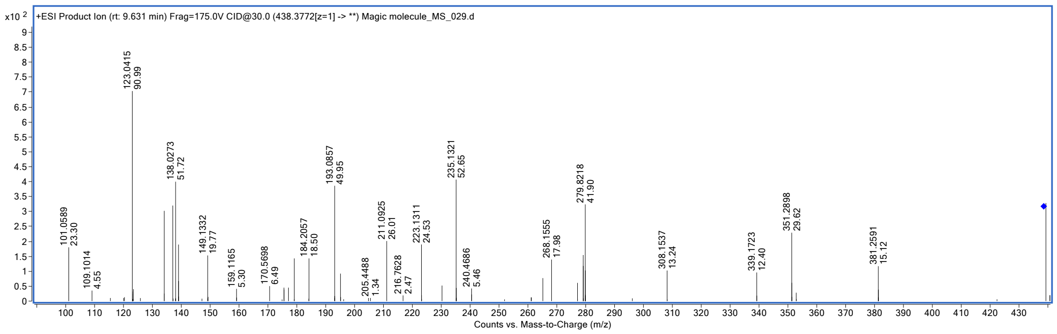

Collision Energy: 50eV

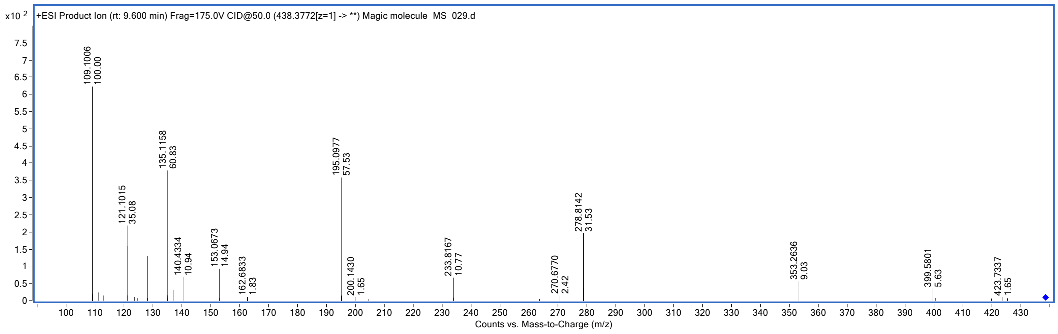

References

1. *Mass Bank of North America (MoNA)*. https://mona.fiehnlab.ucdavis.edu/.

2. Horai, H.; Arita, M.; Kanaya, S.; Nihei, Y.; Ikeda, T.; Suwa, K.; Ojima, Y.; Tanaka, K.; Tanaka, S.; Aoshima, K.; Oda, Y.; Kakazu, Y.; Kusano, M.; Tohge, T.; Matsuda, F.; Sawada, Y.; Hirai, M. Y.; Nakanishi, H.; Ikeda, K.; Akimoto, N.; Maoka, T.; Takahashi, H.; Ara, T.; Sakurai, N.; Suzuki, H.; Shibata, D.; Neumann, S.; Iida, T.; Tanaka, K.; Funatsu, K.; Matsuura, F.; Soga, T.; Taguchi, R.; Saito, K.; Nishioka, T. MassBank: A Public Repository for Sharing Mass Spectral Data for Life Sciences. *J. Mass Spectrom.* **2010**, *45* (7), 703–714. https://doi.org/10.1002/jms.1777.
